# Versatile Metal Phthalocyanine‐Based Memristive Nanowire Network: Unraveling the Dynamics of Digital to Analog Switching

**DOI:** 10.1002/smsc.202500424

**Published:** 2026-01-28

**Authors:** Sudeshna Maity, Aparajita Mandal, Prabhanjan Pradhan, Ankita Ghosh, Dinesh Topwal, Biplab K. Patra, Tapobrata Som

**Affiliations:** ^1^ SUNAG Laboratory Institute of Physics, Sachivalaya Marg Bhubaneswar 751005 India; ^2^ Materials Chemistry and Interfacial Engineering Department CSIR‐Institute of Minerals and Materials Technology Bhubaneswar 751013 India; ^3^ Academy of Scientific and Innovative Research (AcSIR) Ghaziabad 201002 India; ^4^ Homi Bhabha National Institute, Training School Complex Anushakti Nagar Mumbai 400085 India; ^5^ Condensed Matter and Material Physics Group Institute of Physics Sachivalaya Marg Bhubaneswar 751005 India

**Keywords:** artificial synapse, digital switching, impedance spectroscopy, metal phthalocyanine, molecular memristors, multibit, nanowire network

## Abstract

Organic memristors with tunable resistive switching (RS) are promising candidates for brain‐inspired neuromorphic computing. This study reports a self‐assembled organic nanowire network memristor based on copper (II) hexadecafluoro‐phthalocyanine (F_16_CuPc), exhibiting digital, multilevel, and analog switching through compliance current (*I*_CC_) modulation. Current–voltage and impedance analyses reveal that the transition in RS behavior is primarily driven by a shift from trap‐limited to trap‐free space charge‐limited conduction as *I*_CC_ increases. In low *I*_CC_, Ag^+^‐cation migration plays a central role in conduction through redox‐assisted Ag–F/ Ag–π interwire interactions, causing abrupt switching. In contrast, higher allowed injection at high *I*_CC_ enables predominant intrawire current conduction via π–π intermolecular interactions, resulting in a gradual RS transition. The novelty of this work lies in the controlled growth of nanowire structures via self‐assembled 2D molecular stacking, which is key to enabling multifunctionality within a pristine, nanowire network‐based molecular memristive system designed for hybrid digital‐neuromorphic applications. These findings significantly broaden the functional scope of metal phthalocyanine‐based nanowire network architecture, advancing their application toward flexible, energy‐efficient, multifunctional, and wearable smart electronics.

## Graphical abstract

This work demonstrates a memristive, self‐assembled nanowire network based on F_16_CuPc that exhibits digital, multilevel, and analog synaptic‐like switching, achieved simply by tuning the current. The transition, driven by a shift from trap‐limited to trap‐free space‐charge‐limited current conduction, enables modulation from interwire to intrawire pathways, marking the first demonstration of coexisting switching modes in a single organic molecular memristive platform.

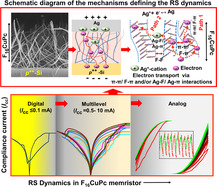

## 1 Introduction

Amidst the rise of artificial intelligence (AI) and the internet of things (IoT), traditional technologies struggle to keep pace with the impending data surge.^[^ ^1^ ^]^ These challenges have sparked an urgent need for innovative devices and systems that offer energy‐efficient, high‐density data storage and parallel computing. Memristors, renowned for their history‐dependent resistive switching (RS) behavior, are the key to unlocking the future of electronics, powering next‐generation platforms like in‐memory and neuromorphic computing with high efficiency.^[^ ^2^ ^]^ Oxide‐based nonvolatile memristors, in particular, have emerged as leading candidates due to their significant commercial potential and superior performance metrics.^[^ ^3^, ^4^, ^5^ ^]^ However, many oxide memristors rely on an electroforming process that typically demands high voltages and currents, leading to considerable power dissipation, potential device damage, and issues such as unpredictable conductance and nonuniformity, which hinder reliable data storage and computation.^[^ ^6^, ^7^, ^8^ ^]^ To overcome these limitations, an intense effort is on to decipher forming‐free memristors having enhanced reliability and performance.^[^ ^9^, ^10^, ^11^ ^]^ In this context, organic materials, including polymers,^[^ ^12^, ^13^ ^]^ donor‐acceptor complexes,^[^ ^14^ ^]^ organic framework materials,^[^ ^15^ ^]^ and small molecules,^[^ ^16^, ^17^, ^18^ ^]^ offer a clear advantage over their inorganic counterparts, due to their ability to harness uniform molecular switching mechanisms.^[^ ^19^, ^20^ ^]^ In addition, organic materials are promising due to their miniaturized dimensions, designable molecular structure, low cost, and ease of processing, especially related to their compatibility with flexible substrates, which allows for a wearable smart neuromorphic system with low energy consumption.^[^ ^21^, ^22^ ^]^ Despite these fascinating features, organic memristors remain less explored, primarily due to the complex structure‐function relationships and stability challenges.^[^ ^23^, ^24^ ^]^

Fundamentally, metallophthalocyanines (MPcs) are well‐known 2D, planar aromatic semiconducting small molecules capable of self‐assembling into stacks through π–π interactions, exhibiting excellent chemical and thermal stability along with unique optical and electrical properties.^[^ ^25^, ^26^, ^27^ ^]^ Some of these versatile conjugated transition metal macrocycles and their derivatives demonstrate electricity‐induced nonvolatile resistance states.^[^ ^28^, ^29^ ^]^ For instance, a random spike in current, indicative of digital switching behavior, is reported in N‐CuMe_2_Pc nanowire‐based memristor.^[^ ^30^ ^]^ On the contrary, analog switching is demonstrated in CuPc and ClCuPc‐based memristive devices.^[^ ^31^, ^32^ ^]^ These differences in the RS behavior are typically governed by the specific switching mechanisms involved in the RS process. Factors, such as presence of the central metal atoms (e.g., Cu, Fe, Zn etc.), functionalization of phthalocyanine ligand (e.g., fluorination and chlorination), and surface structures (e.g., microstructures, nanostructures, nanowires, and nanoribbons) play critical roles in defining the intrinsic performance.^[^ ^26^, ^33^, ^34^ ^]^

This work demonstrates the bipolar, forming‐free nonvolatile RS behavior in a copper(II) hexadecafluoro‐phthalocyanine (F_16_CuPc)‐based memristive architecture, fabricated via a low‐cost, bottom‐up physical vapor transport (PVT) method.^[^ ^35^ ^]^ This technique enables the growth of a self‐assembled nanowire network on a *p* ^++^‐Si (100) substrate. It is widely known that F_16_CuPc, a highly stable, n‐type semiconductor, works as an electron transporting layer, exhibiting relatively high electron mobility (≈0.02 cm^2^ V^−1^ s^−1^) due to significant 2D molecular stacking and π‐electron overlap.^[^ ^36^, ^37^, ^38^ ^]^ Our results reveal how regulation of the compliance current (*I*_CC_) enables manifestation of both digital and analog switching characteristics in the F_16_CuPc molecular memristors. In this context, the coexistence of digital and analog switching in a single device is often reported in inorganic (metal oxides, ferrites, and perovskites) memristors.^[^ ^39^, ^40^, ^41^, ^42^, ^43^, ^44^, ^45^, ^46^ ^]^ To the best of our knowledge, this feature has not yet been systematically investigated in organic memristors, including those based on polymers and small molecules. As a result, this limits their applicability in fully harnessing uniform molecular switching for advanced memory and neuromorphic applications, such as reservoir computing, wherein self‐assembled nanowire switching medium enables in‐materia computing at reduced training cost.^[^ ^47^ ^]^ This work demonstrates that, at low *I*_CC_ (0.1 mA), the device exhibits distinct digital switching behavior. Furthermore, by systematically increasing *I*_CC_ (0.5–10 mA), the nanowire network‐based memristor displays stable multilevel resistive states, highlighting its potential for high‐density multistorage applications. Interestingly, at this stage, the same memristor successfully emulates stable bio‐synaptic behaviors such as potentiation and depression, which is contrasting to the conventional RS dynamics commonly observed in most inorganic memristors.^[^ ^44^ ^]^ To elucidate the observations, the conduction mechanisms for both the compliance‐limited (*I*_CC_ = 0.1 mA) and compliance‐free (beyond 10 mA) regimes are systematically investigated by analyzing current–voltage (*I–V*) characteristics, supported by electrochemical impedance spectroscopy (EIS) studies. The novelty of our approach lies in the controlled growth of nanowire structures via self‐assembled 2D molecular stacking, which plays a critical role in enabling the coexistence of multiple switching phenomena. This work unveils the potential of MPc‐based nanowires to achieve tunable RS behavior within a single device, spanning digital, multilevel, and analog modes, reinforced by a vivid understanding of the RS behavior in such organic small molecule‐based self‐assembled nanowire structures. This versatility paves the way for customized memory and computing applications, while also pointing toward vast opportunities for employing MPc nanowires in fabricating nanodevices and integrated nanoscale circuit architectures. We believe these insights open exciting possibilities for designing low‐cost, robust, small molecule‐based memristive platforms, driving the development of next‐generation, energy‐efficient wearable smart electronics, such as brain‐machine interfaces, robotics, data storage, wearable healthcare devices, and smart textiles.

## 2 Results and Discussion

### 2.1 PVT‐Processed F_16_CuPc Nanowire Network

**Figure 1:**
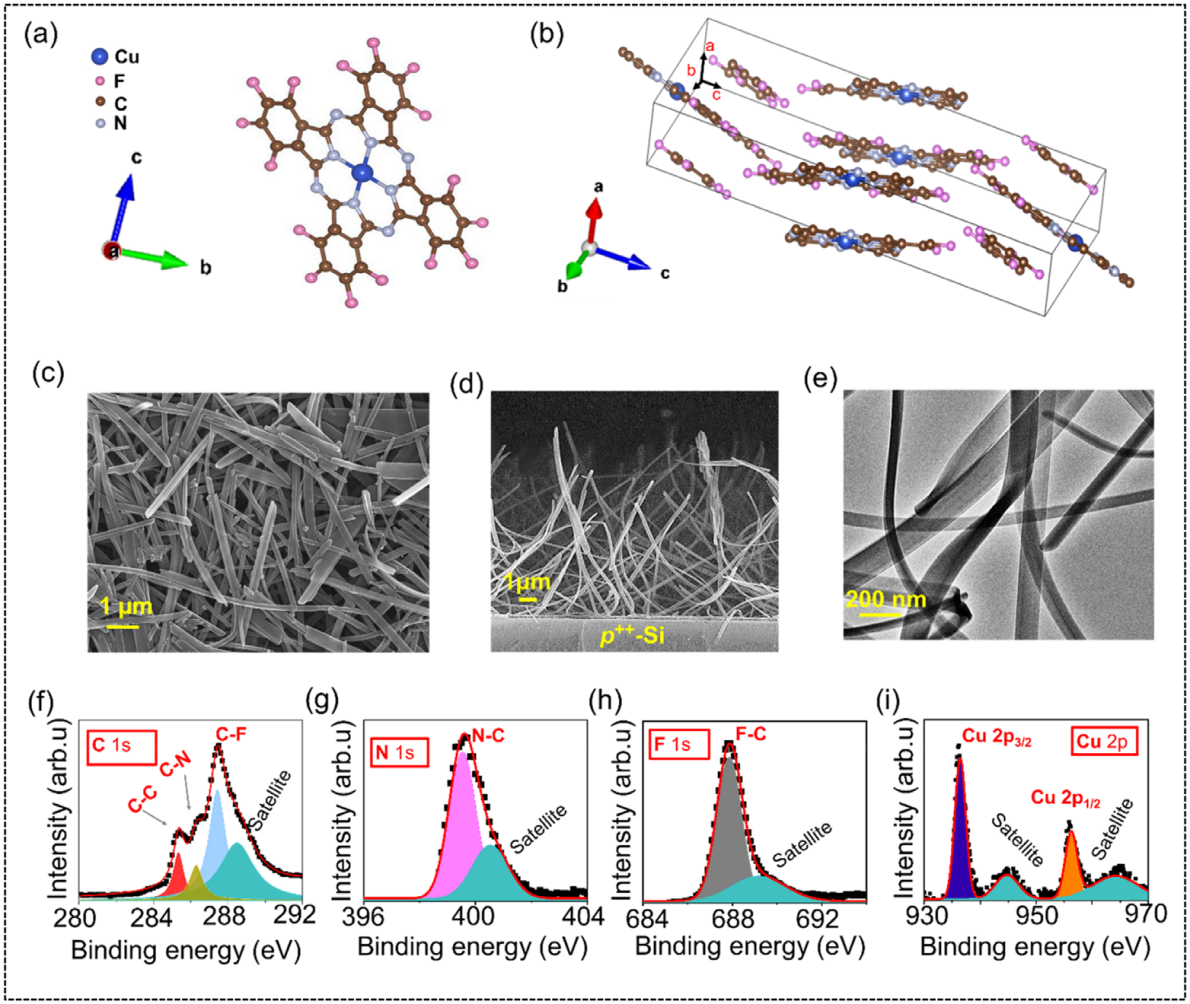
a) Molecular and b) unit cell structure of F_16_CuPc. c) FESEM plan‐view, d) FESEM cross‐sectional view and e) TEM image of PVT‐grown self‐assembled F_16_CuPc nanowire network. High‐resolution XPS spectra of F_16_CuPc nanowire network centered on f) C 1*s*, g) N 1*s*, h) F 1*s*, and i) Cu 2*p*.

**Figure** **1**a,b present the chemical structure of the disk‐shaped F_16_CuPc molecule and a representative unit cell showing the layered molecular stacking in all three directions, respectively. Notably, in MPcs, the size of the central metal atom (here, Cu) determines the intermolecular spacing along the *a*‐axis (Figure 1b).^[^ ^48^ ^]^ Figure 1c presents the plan‐view image of the PVT‐grown F_16_CuPc sample using field emission scanning electron microscopy (FESEM), confirming the formation of densely packed interconnected nanowire networks. Figure S1, Supporting Information presents an image of the as‐grown bare film.

It is important to mention here that the dimensionality of the F_16_CuPc crystal can be controlled by adjusting key reaction variables, such as precursor temperature, substrate temperature, carrier gas flow rate, and deposition time.^[^ ^26^, ^35^, ^48^, ^49^, ^50^ ^]^ It has been observed that an increase in substrate temperature beyond ≈200 °C often results in nanowire formation. It is well known that F–π intermolecular interaction between alternative packing of F_16_CuPc molecules along the *b* and *c*‐axis determines the width and thickness of the nanowires, respectively, whereas, π–π interactions along the *a*‐axis is much stronger than the interaction forces along the *b*‐and *c*‐axis, therefore defining the length of the wires. The undertaken PVT process (precursor temperature ≈495 °C, substrate temperature ≈250 °C, gas flow rate ≈45 sccm) results in nanowire structures, as evident from the cross‐sectional FESEM image in Figure 1d, highlighting the vertical growth of the nanowires on the *p* ^*++*^‐Si substrate. For a clearer view of the nanowires, a plan‐view transmission electron microscopic (TEM) image of the F_16_CuPc nanowire network has also been presented in Figure 1e, emphasizing that the width of the wires ranges from several tens to a few hundreds of nanometers. The purity of the crystal phase is investigated by performing X‐ray diffraction (XRD) measurement on as‐prepared F_16_CuPc sample. As shown in Figure S2, Supporting Information the XRD pattern displays a strong peak centered at 2*θ* = 6.30° is related to the (002) plane, indicating equally spaced layered structure formation.^[^ ^35^, ^48^, ^50^ ^]^ The nature of the chemical bonds in the as‐prepared sample is investigated using the core level X‐ray photoelectron spectroscopy (XPS), as shown in Figure 1f–i. It is evident that the C 1*s* spectra of the F_16_CuPc films are reported to display at least three different characteristic peaks.^[^ ^51^ ^]^ As shown in Figure 1f, the high binding energy peak located at 287.4 eV with a small shoulder can be assigned to the carbon‐fluorine (C—F) bonding due to a strong charge transfer from C to F atomic sites. The peak at 286.3 eV is due to the eight pyrrole C atoms, and the other at 285.3 eV is for eight aromatic C atoms in the benzene rings, which are not bound to F atoms. The N 1*s* core level spectrum possesses a main component at 399.5 eV, which represents the N—C bonding with a π–π* satellite feature at high binding energy (Figure 1g). The F 1*s* peak contains only one component from the C—F bonding at 687.8 eV with a small shoulder (Figure 1h). The Cu 2*p* spectrum is characteristic of the so‐called Cu (II), which includes two strong peaks as is evident from Figure 1i. Cu 2*p*_3__/2_ peak is located at a binding energy of 935.7 eV, whereas the Cu 2*p*_1__/2_ is at 955.6 eV. Importantly, the observation from XPS measurement fairly aligns with the literatures, indicating the coordination of the core copper to nitrogen atoms in phthalocyanine ligands remains intact during the PVT growth process undertaken in our study.^[^ ^52^ ^]^ Energy dispersive x‐Ray (EDX) spectrum is further carried out to confirm the presence of constituent elements. Figure S3, Supporting Information presents the EDX mapping, corroborating the uniform elemental distribution of C, F, N, and Cu along the scanned region.

### 2.2 RS Performance

#### 2.2.1 Digital Switching

**Figure 2:**
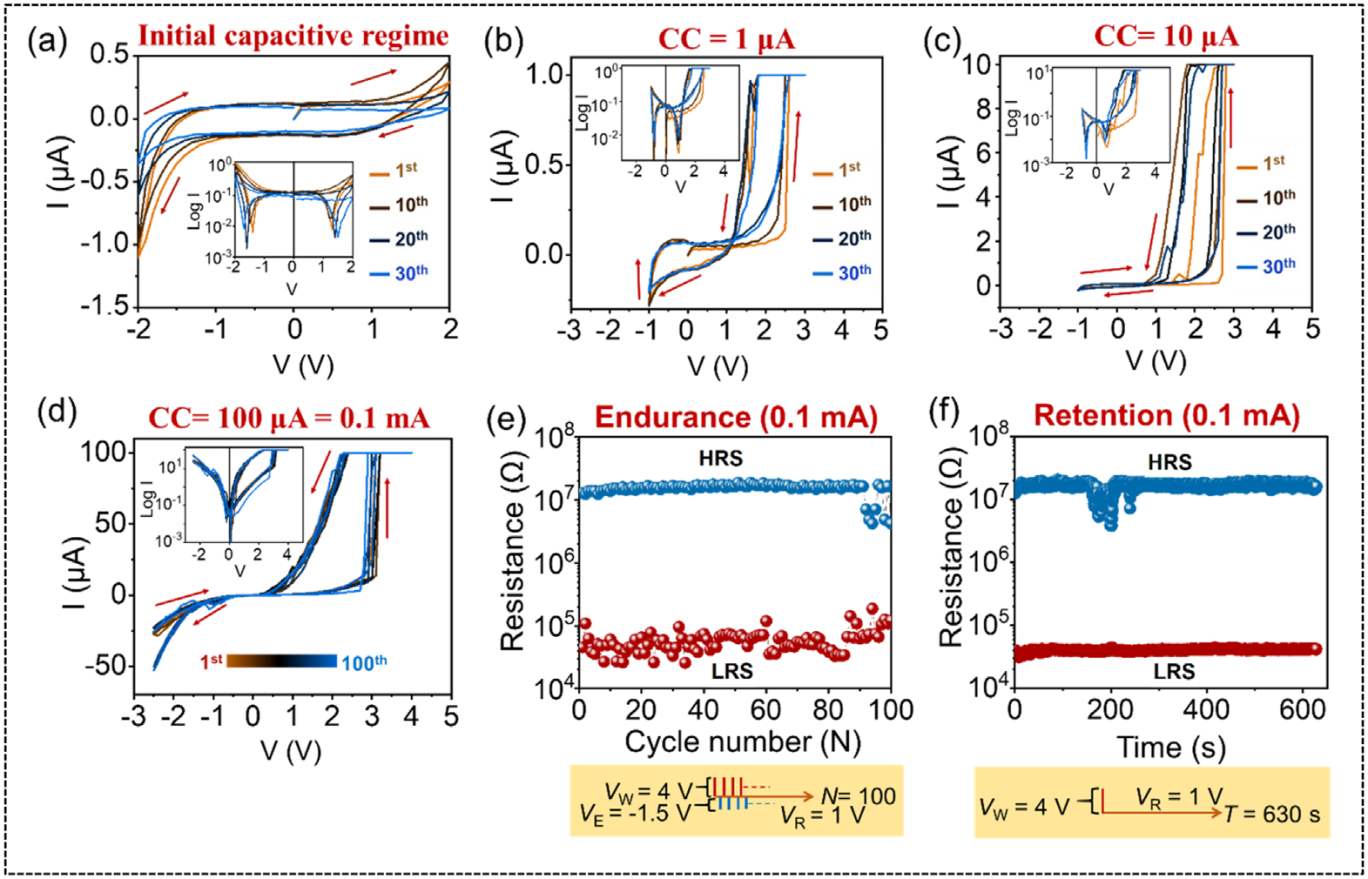
The *I–V* characteristics of the *p* ^++^‐Si/F_16_CuPc/Ag memristor. a) Initial capacitive regime (0 V → 2 V → −2 V  →  0 V). Digital switching at b) 1 μA (0 V → 3 V → −1 V  →  0 V), c) 10 μA (0 V → 3 V → −1 V → 0 V) and d) 100 μA (0 V → 4 V → −2.5 V → 0 V) with semilogarithmic *I–V* characteristics in the insets. e) Endurance and f) memory retention at 100 μA compliance current (CC) with the structure of the given voltage inputs.

The *I* *–* *V* characteristics of the memristor having configuration: *p* ^++^‐Si/F_16_CuPc/Ag is measured under various *I*_CC_ by applying external voltage sweeps. **Figure** **2** shows that the *I* *–* *V* characteristics are significantly altered by tuning *I*_CC_ values. At the initial stage, while applying DC sweep bias following the sequence of 0 V → 2 V → −2 V → 0 V, the *I* *–* *V* shows a nonzero crossing capacitive behavior for consecutive sweep cycles as shown in Figure 2a. This capacitance effect is also observed in FTO/CuPc/Au memristor as documented in a recent report by Liao and coworkers.^[^ ^53^ ^]^ In fact, combining the capacitance effect with RS behavior is highly beneficial for designing new, versatile devices.^[^ ^54^ ^]^ It is also clear from the Figure 2a that the capacitance effect does not possess any oxidized and reduced peaks despite the *I* *–* *V* being measured under ambient conditions (average relative humidity (RH) ≈ 40%), thus eliminating any influence of humidity in the *I* *–* *V* curves.^[^ ^55^ ^]^ Furthermore, the recorded XPS spectrum on the specimen does not show any noticeable signature of oxygen (O 1s) within the detection limit (Figure S4, Supporting Information). Henceforth, the role of oxygen adsorption can also be excluded in obtaining the observed RS hysteresis behavior, which will be discussed later. In contrast, Li et al. shows the appearance of O 1s peak at 531.5 eV, while F_16_CuPc samples are annealed under 90% RH due to the formation of hydroxyl and carbonate species.^[^ ^56^ ^]^ Next, by modifying the DC sweep bias following the sequence of 0 V → 3 V → −1 V → 0 V and applying *I*_CC_ of 1 and 10 μA, distinct RS behaviors are observed as depicted in Figure 2b,c, respectively. In both the above cases, the memristor exhibits a rapid transition from a high resistive state (HRS) or OFF state to a low resistive state (LRS) or ON state. In this quest, by modifying the DC sweep bias range further to 0 V → 4 V → −2.5 V → 0 V and increasing the *I*_CC_ to 100 μA (0.1 mA), the stability of the RS curve significantly improves (Figure 2d). It is worth mentioning here that the nonzero crossing feature is still present in all the RS curves, as seen from the semilog *I* *–* *V* characteristics in the insets of respective figures.

This result demonstrates a reproducible digital RS window at a *V*_SET_ of 3 V and a *V*_RESET_ of −1 V in the F_16_CuPc nanowire network‐based molecular memristor. The endurance or reversibility of the associated memory is examined using a voltage pulse sequence known as “Write‐Read‐Erase‐Read”. As presented in Figure 2e, a sequence of 100 identical pulses with a width (Δ*T*) of 500 ms is applied to “Write” at *V*_W_ = 4 V (i.e., driving the device into LRS) and then “Erase” at *V*_E_ = −1.5 V (i.e., returning back into its HRS). The respective states are probed under the read voltage, *V*_R_ = 1 V. It is clearly noticed that the current levels are well separated over 100 cycles, maintaining an impressive ON/OFF ratio (*I*_LRS_/*I*_HRS_ = *R*_HRS_/*R*_LRS_ ≈ 408.9). Importantly, it is observed that continuous application of 1 V without prior programming does not induce RS or artificially sustain the LRS, ensuring that the observed characteristics are intrinsic to the device. To further validate the nonvolatile memory characteristics, retention tests are conducted under identical programming conditions by applying a write pulse (*V*_W_ = 4 V, Δ*T* = 500 ms) followed by read operations at *V*_R_ = 1 V over an extended period (Figure 2f). Both the HRS and LRS show negligible degradation for at least 630 s, demonstrating stable charge retention and minimal relaxation effects. These results fortify that the present F_16_CuPc‐based molecular memristor offers an excellent information storing capability.^[^ ^57^ ^]^

#### 2.2.2 Multilevel Switching

**Figure 3:**
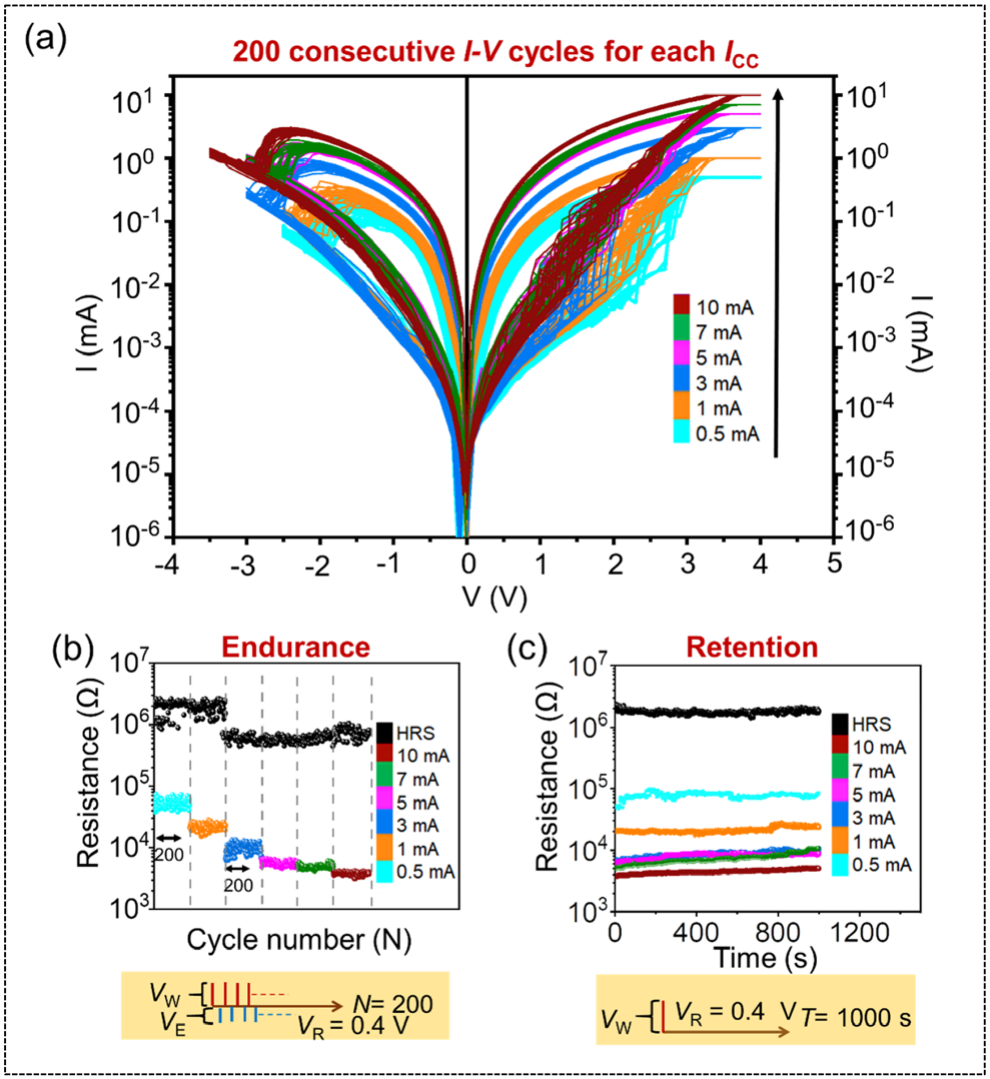
Multilevel switching in *p* ^++^‐Si/F_16_CuPc/Ag memristor at different *I*_CC_ (0.5, 1, 3, 5, 7, and 10 mA) a) Semilog *I–V*, b) endurance, and c) retention with the structure of given voltage inputs. Colors in the palette represent different *I*_CC_.

**Figure 4:**
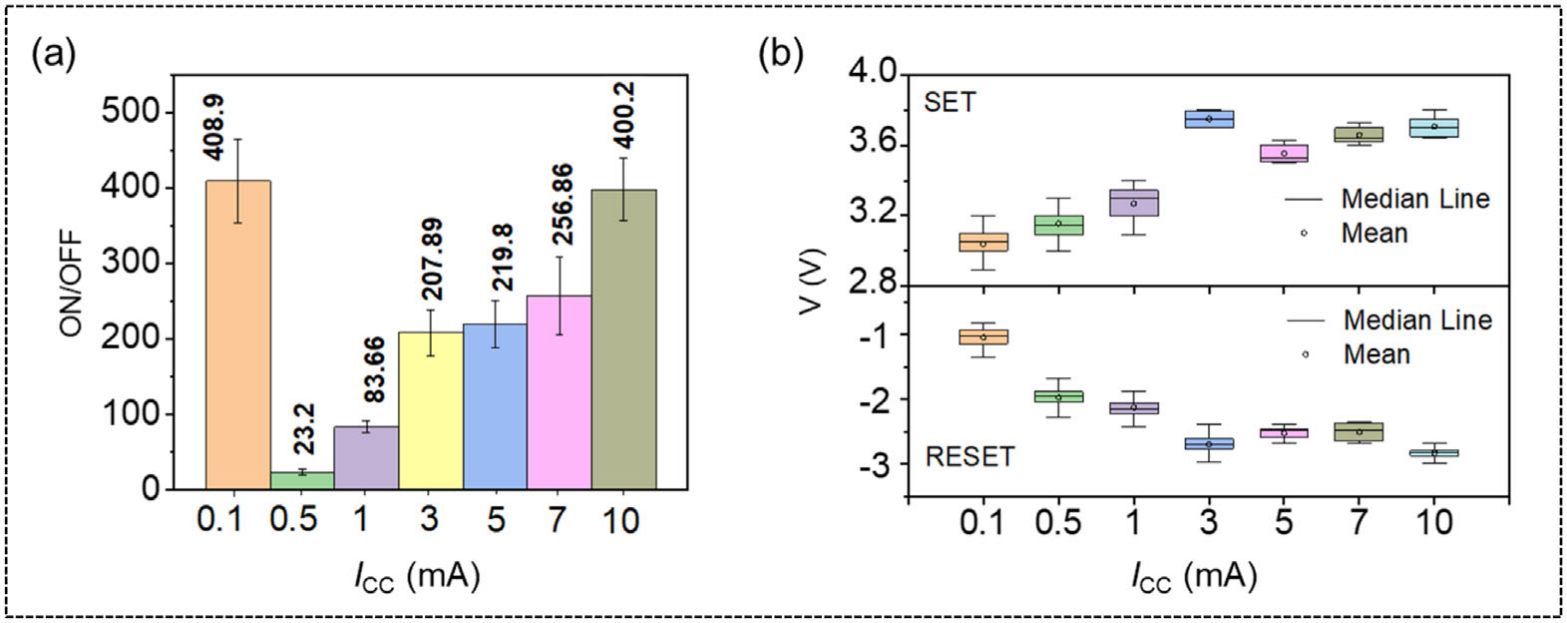
a) ON/OFF ratio and b) statistical distribution of *V*_SET_ and *V*_RESET_ as a function of *I*_CC_.

The potential of present molecular RS device is further explored by varying the *I*_CC_ beyond above discussed limits. For instance, multiple low resistance states can be induced by a gradual increase in *I*_CC_ from 0.5–10 mA, as displayed in **Figure** **3**a, wherein the distinct color in the palette represents the various *I*_CC_ values. The *I* *–* *V* characteristics are presented on a semilogarithmic scale to clearly illustrate the multilevel behavior, demonstrating six distinct memory levels. Further increase in *I*_CC_ results in compliance‐free regime, as depicted in Figure S5, Supporting Information. Importantly, it can be noticed that in the reverse direction, the negative differential resistance (NDR) effect, with an abrupt fall in current, becomes more apparent with increased *I*_CC_. This suggests a possible contribution of Joule heating in attaining the RESET state of the device.^[^ ^58^ ^]^ Specifically, the low thermal conductivity of organic semiconductors resists rapid heat dissipation, thus sensitive to Joule heating while operating under high current.^[^ ^59^ ^]^ This consideration fairly explains the unclear RESET process in digital mode, as heating is not enough at such a low current (≤0.1 mA). It is noteworthy that the observed RS behavior is independent of the order of the *I*_CC_ chosen for the measurement, which again reflects the reproducibility of the switching mechanism. Also, by choosing a random *I*_CC_ for measurement (here, 0.5 → 3 → 1 → 5 → 10 → 7 mA) one can avoid potential artifacts due to increasing electrical stress. Additionally, the capacitive feature is observed to be disappeared while *I*_CC_ is as high as 5 mA (Figure S6, Supporting Information). Furthermore, endurance over 200 successive cycles makes the molecular memristor suitable for applications requiring efficient data encoding and processing (Figure 3b). It is also important to note that the transitions between different resistive states are reproducible, indicating their potential use in rewritable or random‐access memory (RAM) applications, where reliable and rapid state changes are essential for data storage and retrieval.^[^ ^60^ ^]^ Figure 3c demonstrates memory retention ability stable for more than 10^3^ s, thus highlighting the reliability of the memristor in maintaining its multiple states over an extended period of operations. Notably, the minor fluctuations observed in the HRS and LRS memory states fall within the acceptable range of stochasticity inherent to any memristive phenomena.^[^ ^61^ ^]^ The ON/OFF ratio is observed to have sharply dropped to ≈23 at 0.5 mA, compared to the ON/OFF ratio at 0.1 mA, as clearly illustrated in **Figure** **4**a. This is due to the reduced gap between the ON and OFF states, as HRS resistance is seen to be decreased by approximately one order of magnitude compared to the HRS resistance at 0.1 mA (Figure 2e and Figure 3b).

As the *I*_CC_ increases to 10 mA, the LRS resistance progressively decreases, resulting in the ON/OFF ratio recovering to ≈400. This observation also indicates that once the allowed current through the memristor increases, a modulation in current conduction is expected (as discussed later). Figure 4b presents the mean and standard deviations of *V*_SET_ and *V*_RESET_ across the range of *I*_CC_ studied for digital and multilevel modes. It is found that in parallel to the rise in *I*_CC_ from 0.1–10 mA, both *V*_SET_ and *V*_RESET_ show a slightly increasing trend in the positive and negative direction, respectively. The small variations in the *V*_SET_ and *V*_RESET_, with standard deviations ranging from ±0.04 – ±0.16, make the F_16_CuPc nanowire network‐based memristors suitable for applications requiring precise voltage control at different current settings. This result highlights important insights into the consistency and reliability of implementing multilevel memory not only to break the limitation of low‐density memory but also link to binaries.^[^ ^62^, ^63^, ^64^ ^]^

#### 2.2.3 Analog Switching

**Figure 5:**
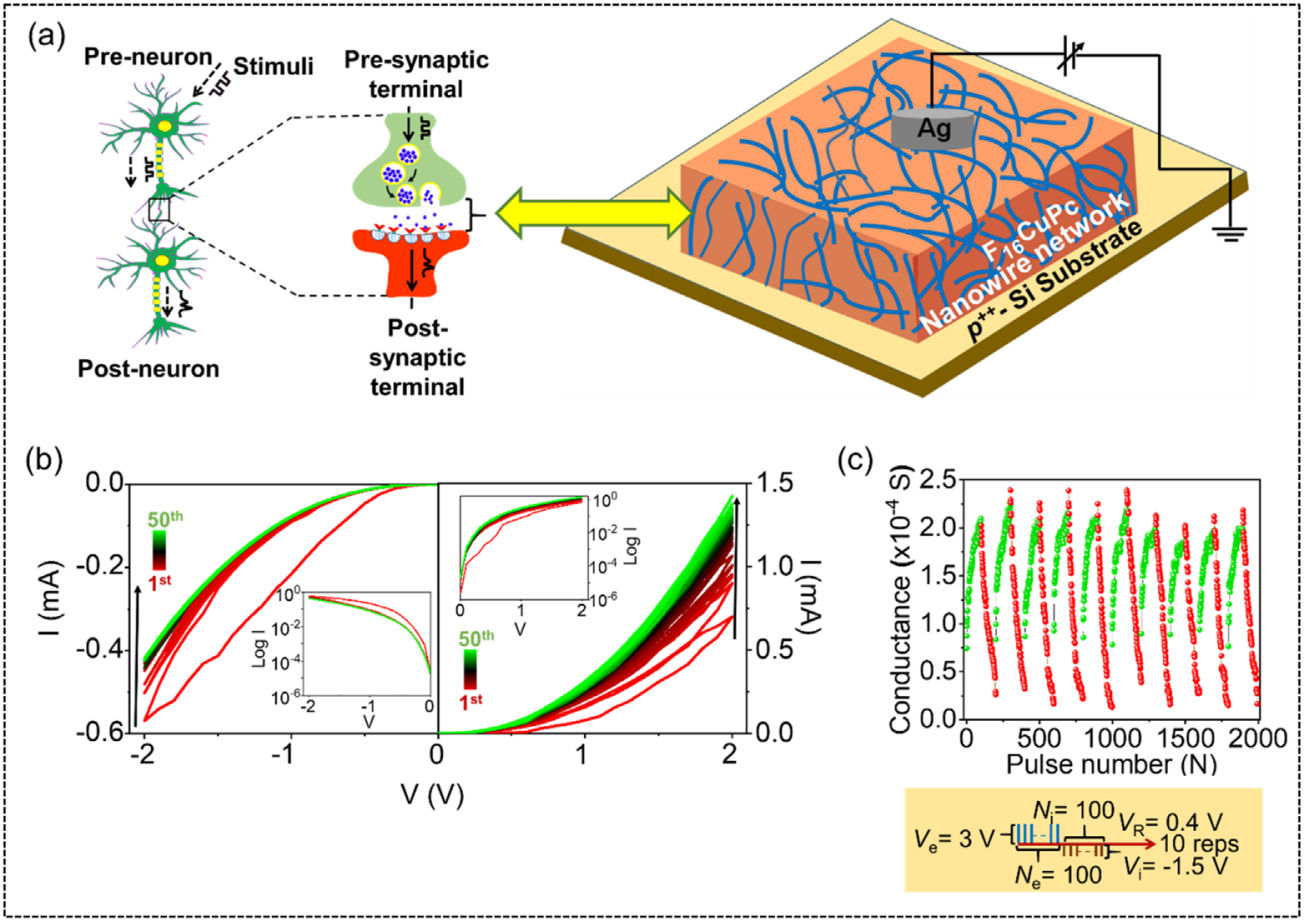
a) Schematic diagram of biological neurons and a synapse, and its analogy with a memristor‐based two‐terminal electronic synapse (*p* ^++^‐Si/F_16_CuPc/Ag). b) Analog SET under 50 consecutive positive sweeps (0 V → 2 V → 0 V) and analog RESET under 50 consecutive negative sweeps (0 V → −2 V → 0 V) with the semilogarithmic *I*–*V* characteristics in the insets. c) Potentiation and depression behavior of the memristor with the structure of given voltage input.

The connectivity of a memristor can be dynamically modulated by adjusting its conductance through voltage pulses, analogous to neural spikes. This enables effective emulation of biological synaptic behavior, wherein the top and bottom electrodes function as pre‐ and postsynaptic terminals according to the direction of applied bias. The schematic presentation of signal transmission in biological synapses through synaptic weight change is presented in **Figure** **5**a. The strength of the connectivity between two artificial synaptic terminals can be regulated by adjusting their conductance. Notably, the multilevel switching study reveals a progressively more gradual SET process with increasing *I*_CC_ (Figure 3a), indicative of an activation process prior to the analog switching, analogous to the electroforming process that typically precedes conventional digital switching. However, this aspect has not been properly acknowledged and emphasized in the existing literature. Here, the same memristor enables exhibiting a typical analog SET and RESET behavior with gradually increasing and decreasing hysteresis windows under 50 consecutive positive forward sweeps (0 V → 2 V)/negative reverse sweeps (0 V → −2 V), respectively (Figure 5b). This new switching behavior is notably distinct from the former digital mode. In this context, it is worth noting that the coexistence of analog and digital switching within a single device is a well‐reported phenomenon in inorganic memristors. For example, a very recent report by Yao et al. demonstrates oxygen vacancy‐controlled digital RS to Ag conductive filament‐based analog RS conversion in the Y_3_Fe_5_O_12_ ferrite‐based devices.^[^ ^39^ ^]^ Similar observations are reported by Mao et al.^[^ ^40^ ^]^ and Li et al.^[^ ^43^ ^]^ in metal oxide‐based FTO/δ‐MnO_2_/Ag and Si/SiO_2_/Ti/Pt/NiO/Ag memristors, respectively. Zhu et al. demonstrates the same observation in CsPbBr_3_ perovskite‐based memristive structure.^[^ ^45^ ^]^ This phenomenon, however, remains unexplored in organic memristors to date. Figure 5c demonstrates typical potentiation and depression behavior, making the device advantageous for synaptic applications. The memristor exhibits good linearity over 10 repetitions with a series of 100 identical excitatory stimuli (*V*_e_ = 3 V, Δ*T* = 200 ms), followed by another series of 100 identical inhibitory stimuli (*V*_i_ = −1.5 V, Δ*T* = 200 ms), leading to increasing conductance, i.e., potentiation and decreasing conductance, i.e., depression, respectively. The wide resistance tuning range of the F_16_CuPc molecular memristors indicates their potential to emulate biological synaptic plasticity, which plays a crucial role in learning and memory processes in the brain.^[^ ^65^ ^]^ This adaptability also highlights the potential of metal phthalocyanine‐based organic small molecule devices as attractive options for implementing advanced wearable neuromorphic systems, enabling more complex and efficient computing capabilities.^[^ ^66^ ^]^ Table S1, Supporting Information presents a state‐of‐the‐art performance summary of metal phthalocyanine‐based memristors, highlighting the novelty and significance of the present study, exhibiting digital and analog RS in a single device.

### 2.3 Conduction Mechanism

In order to shed light on the switching behaviors discussed above, a thorough understanding of the underlying charge transport processes within the device structure is essential. The charge conduction mechanisms are elucidated during the SET process for both the compliance‐limited (0.1 mA) and compliance‐free (beyond 10 mA) RS regime. The *I* *–* *V* curves in HRS and LRS are replotted in a double‐logarithmic scale and analyzed using the linear fitting methods, from which the corresponding slope values are extracted.

**Figure 6:**
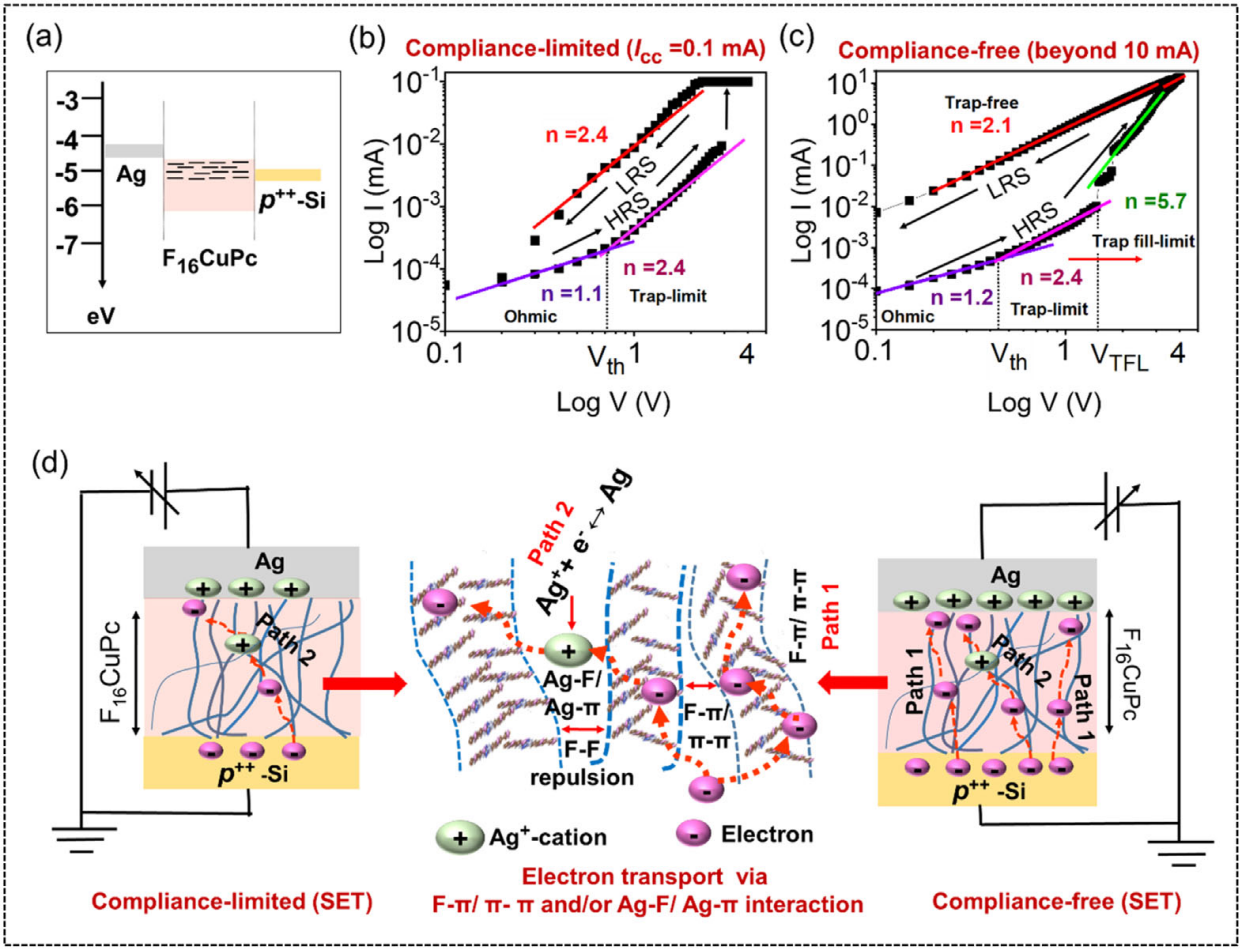
a) Energy band diagram of the *p* ^++^‐Si/F_16_CuPc/Ag memristor. Charge conduction mechanism in the SET process for b) compliance‐limited and c) compliance‐free regime. Solid lines represent linear fits to double‐logarithmic *I–V* plots. d) Schematic illustration of the conduction mechanism in F_16_CuPc self‐assembled nanowire network‐based memristor. Blue lines represent F_16_CuPc nanowires, red dotted arrows indicate conduction paths.

Under the compliance‐limited RS regime during HRS, initially a linear relationship is observed at the low‐voltage region (*V* < 1 V) (**Figure** **6**b). This indicates ohmic conduction (*J*_Ohmic_ ∝*V* ^n ≈ 1^, n is the exponent), where the current is primarily carried by thermal carriers due to weak injection from the electrodes. Next, with the increase in applied bias above a threshold voltage (*V*_th_ in Figure 6b), space charge‐limited current (SCLC; *J*_SCLC_ ∝*V* ^n ≈ 2^) emerges, where the injected carrier concentration exceeds that of the thermally generated one. Intrinsically weak intermolecular bonds in (n‐type) organic semiconductors favor the formation of (acceptor‐like) defect/trap levels with a broad energy distribution within the bandgap (Figure 6a), which localizes a large proportion of charge carriers.^[^ ^67^ ^]^ These trapped charges in the semiconductor play a dominant role in generating the hysteresis effect.^[^ ^68^ ^]^ In the compliance‐limited RS regime, at higher voltages (*V* > 1 V), the trap‐limited SCLC dominates the HRS and the entire LRS (Figure 6b). On the contrary, the conduction mechanism for the compliance‐free RS regime shows a clear transition from the trap‐limited SCLC to the trap fill‐limited current (TFLC) conduction due to increased free carrier density from injection at *V* = *V*_TFL_ (Figure 6c).^[^ ^67^ ^]^ At this stage, the quasistatic Fermi level shifts up above the energy of the shallowest traps, thus all the traps get filled,^[^ ^69^ ^]^ resulting in a significant increase in current (n ≈ 6). The TFLC is the intermediate condition for the transition from the trap‐limited SCLC to the trap‐free SCLC. Consequently, the current in the higher voltage region in HRS and the entire LRS process exhibits child's law dependent (n ≈ 2) trap‐free conduction, indicating that trapped electrons are not further released, thus the sample behaves as free of traps, providing an easy path for bulk‐mediated conduction. The cycle‐to‐cycle and device‐to‐device tests are also performed to verify the extracted slope values (Figure S7–S10, Supporting Information), validating the reproducibility of the slope‐based analysis.

In other words, under a compliance‐limited regime, RS is governed by the trap‐limited SCLC, whereas the compliance‐free RS regime is characterized by trap‐free SCLC, which is driven by enhanced injection at a higher allowed current. We previously excluded the influence of humidity and oxygen in obtaining the RS hysteresis. Here, we propose that upon attaining a critical threshold, the formation of space charge and migration of Ag^+^‐cations through the interwire voids becomes dominant, influencing the charge trapping dynamics in F_16_CuPc. The diffusion and migration of Ag^+^‐cations across the phthalocyanine mesh‐like structure has already been reported by earlier studies.^[^ ^30^ ^]^ Fundamentally, the charge carriers can efficiently traverse the device through two main pathways: 1) intrinsic trapping and detrapping through intrawire π–π interactions (see path 1, Figure 6d) and 2) redox‐assisted charge interaction between adjacent Ag^+^‐cation and the fluorinated‐phthalocyanine ring through interwire paths (Ag–F/Ag–π interactions, path 2, Figure 6d). In the latter case, the Ag^+^‐cation acts as a transient charge reservoir, which effectively shortens the charge‐transport pathway.^[^ ^50^, ^51^, ^70^ ^]^ It is important to mention here, in either cases the charge transfer via F–π interaction is more feasible, since the F–F interactions are largely repulsive, contributing very little to conduction. Having fluorine, as a highly electronegative peripheral atom in the molecular nanowire, inherently supports the proposed conduction path, where both mechanisms involve a reversible reduction of central Cu(II) to Cu(I).^[^ ^51^ ^]^ In the compliance‐limited regime at low injection levels, migrated Ag^+^‐cations play a central role in completing the conduction pathway through interwire interactions (see path 2, Figure 6d), which also facilitates direct F–π/ π–π conduction between wires. This process enhances the otherwise poor current conduction of the device, resulting in an abrupt increase in current. However, this mechanism leads to comparatively less stable RS characteristics due to Ag^+^‐cation involvement, causing visible fluctuations between ON/OFF states, as evident in Figure 2e. On the other hand, in a compliance‐free regime, higher allowed injection at high *I*_CC_ enhances injected carrier density, enabling predominant current conduction along the nanowires itself (see path 1, Figure 6d), which is consistent with bulk‐mediated trap‐free SCLC observed in *I* *–* *V* characteristics (Figure 6c). In this regime, the integrated contributions from multiple coexisting conduction pathways, primarily through the nanowires and their π‐coupled interlinked network, along with minor contributions from Ag^+^‐filled interwire channels, result in a more gradual and stable RS transition.^[^ ^39^, ^71^, ^72^ ^]^ Notably, improved reproducibility between ON/OFF states with rising *I*_CC_ also indicates dominance of intrawire π–π interaction under high current conditions (Figure 3b). Furthermore, as reflected in Figure 5c, a highly gradual potentiation/depression behavior validates the bulk‐mediated charge trapping/de‐trapping instead of Ag^+^‐mediated, which is prevalent in higher *I*_CC_. In contrast, an overall stochasticity in potentiation/depression acquired at low *I*_CC_ (Figure S11, Supporting Information) reinforces the conclusions drawn from the *I* *–* *V* analysis, indicating Ag–F/Ag–π conduction (path 2, Figure 6d). As mentioned earlier, F_16_CuPc is an n‐type aromatic semiconductor whose π–π overlap within its conjugated molecular stacking facilitates charge transport by delocalizing π‐electrons between adjacent molecules. Fundamentally, any structural reorientation or conformational changes affect the electronic coupling strength through the grain boundaries, thereby altering the intermolecular charge‐transfer rate. Correlating the *I* *–* *V* characteristics across different current ranges, we infer that at higher allowed injection under elevated *I*_CC_, enhances trapping of electrons at sites of conjugational distortions. This, in turn, modifies the electrostatic interaction along the stacking axis, establishing rearrangement in π–π stacking along preferred crystal orientations.^[^ ^73^ ^]^ As a direct consequence, a reduced reorganization energy and enhanced charge‐transfer integral between adjacent molecules can be expected.^[^ ^74^ ^]^ The novelty of our approach lies in the controlled growth of nanowire structures via self‐assembled 2D molecular stacking, which inherently promotes a stable trap distribution and facilitates cohesive π–π charge transport. These ordered and stable trap arrays are likely a dominant factor enabling reproducible potentiation and depression over 10 consecutive cycles. Notably, this behavior is realized without requiring composites, interface engineering or the fabrication of quantum well‐like heterojunctions to modulate trap‐controlled conductance.^[^ ^19^, ^75^ ^]^

**Figure 7:**
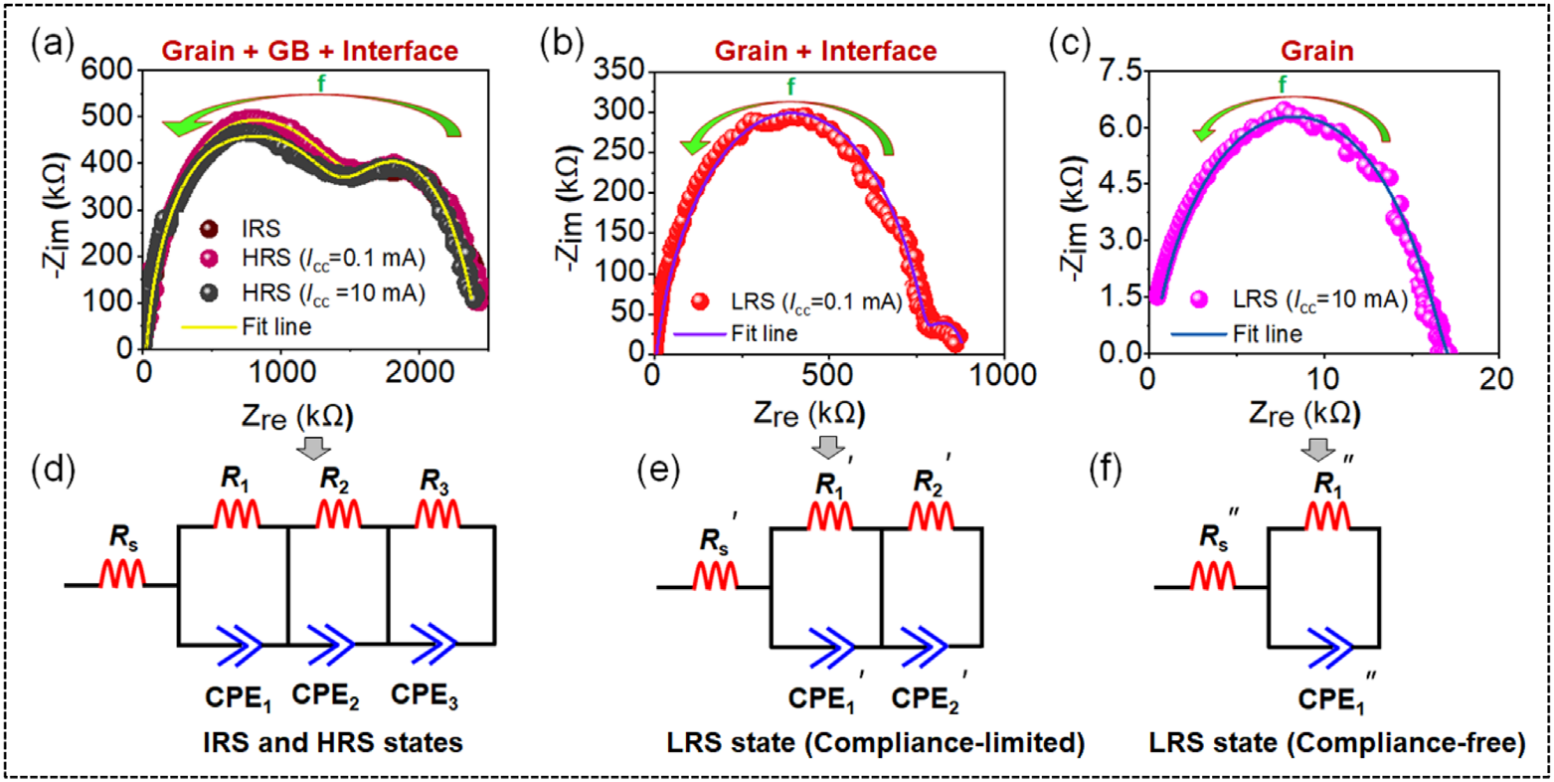
The electrochemical impedance spectra for the *p* ^++^‐Si/F_16_CuPc/Ag memristor. Nyquist plots at a) IRS/HRS (GB stands for grain boundary) and b,c) LRS for the compliance‐limited and compliance‐free regimes, respectively. The fitted curves (solid lines) are achieved using Powell's algorithm. d–f) The equivalent circuits used for fitting the HRS and LRS (compliance‐limited and compliance‐free) curves.

In order to corroborate the conduction mechanisms discussed above, an EIS study is employed at HRS, as well as taking the devices to LRS under compliance‐limited and compliance‐free regimes. It is noteworthy that EIS study has previously been employed to disentangle the various conduction contributions, governing RS hysteresis in inorganic and perovskite‐based memristors.^[^ ^76^, ^77^ ^]^ **Figure** **7**a–c depict the Nyquist plots (symbols) at each of its resistance states; the first initial resistance state (IRS, i.e., virgin state before applying any bias), followed by a transition of the device to the LRS state by applying suitable SET voltages for both regimes. The curved arrow represents the direction of increasing frequency *(f)* from 0.1 Hz– 1 MHz. It is evident from these figures that at LRS both the imaginary (Y‐axis) and real (X‐axis) impedances are significantly lower compared to the HRS, reflecting a clear RS transition. It is also important to highlight that EIS spectra are sequentially obtained by taking the device back to the HRS by applying the desired RESET voltage after each SET process, which closely align with its virgin state, hence showing the repeatability of the measurements (Figure 7a). However, the extent of structural reorientation in the molecular stacking, depending on the magnitude, duration, and sequence of the applied external bias stress, as well as the resulting current, whether reversible or irreversible, cannot be ruled out.^[^ ^59^, ^78^ ^]^ This correlates with the observed 10‐fold reduction in HRS resistance as *I*_CC_ increases from 0.1–0.5 mA (Figure 2e and Figure 3b). The respective fitted curves (solid lines) are obtained using suitable equivalent circuits as presented in Figure 7d–f. The obtained values of equivalent circuit components for all resistance states are presented in Table S2, Supporting Information, where the constant phase element (CPE) represents an imperfect capacitor used in highly disordered systems like organic semiconductors.^[^ ^79^ ^]^ To verify reproducibility of the results from impedance spectroscopy, additional measurements are performed on an independent device under identical experimental conditions across multiple cycles, which show excellent agreement with the main device presented in the manuscript (Figure S12–S14, Tables S3–S5, Supporting Information).

The impedance due to CPE can be defined by two parameters, *Q* and *α*, is expressed by the following equation:

(1) $$Z_{CPE}=\frac{1}{\left[Q{\left(j\omega \right)}^{\alpha }\right]}$$

where *Q* is expressed in units of frequency‐independent capacitance component, *α* is the exponent, and *ω* is the angular frequency (*ω* = 2π*f*). The CPE is identical to a capacitance (*C*) component when the exponent *α* ≈ 1, to a resistance (*R*) component when *α* ≈ 0. Importantly, all the HRS, including IRS spectra, comprise of the signature of three arcs at three different frequency ranges, (I) the low frequency region ≈0.1 Hz–10 Hz, (II) the intermediate frequency region ≈10 Hz–10 kHz, and (III) the high frequency region ≈10 kHz–1 MHz. Accordingly, the equivalent circuits for HRS consist of three Randles circuits (*R* || CPE) that are in series with a pure resistance (*R*_S_), as shown in Figure 7d. The high and intermediate‐frequency arcs can be considered as the contribution from the grains and grain boundaries, respectively, of the molecular nanowire network.^[^ ^80^ ^]^ The third arc at the low‐frequency represents the impedance due to the electrode‐molecule interface, which is related to the charge accumulation.^[^ ^81^ ^]^ The series resistance, *R*_S_ can be attributed to the contact resistance during the charge injection from the electrodes. In contrast to the HRS state, the LRS states under a compliance‐limited regime display an apparent signature of two arcs. One at high and another at low frequency confirms contribution from grains and interfaces, respectively (Figure 7b). Notably, the persistent presence of the interface arc is in good agreement with the existence of nonzero crossing capacitive feature in digital *I* *–* *V* curves, attributing significant charge accumulation at the interface due to the low allowed injection. Under this regime, the complete disappearance of the intermediate arc indicates that the role of the grain boundary becomes negligible, corroborating the findings from *I* *–* *V* measurements. This result is attributed to the involvement of migrated Ag^+^‐cation fillers, which reduce the resistive contribution, mainly associated with repulsive F–F interactions at the grain boundaries by bridging the gaps between nanowires through Ag–F/ Ag–π interactions (path 2, Figure 6d).^[^ ^82^, ^83^ ^]^ Figure 7e presents the corresponding equivalent circuit consists of two Randles circuits that are in series with *R*_S_′. On the contrary, under the compliance‐free regime, the presence of a single arc substantiates grain‐dominated conduction (intrawire π–π interaction) in the LRS, affirming bulk‐mediated trap‐free SCLC conduction (Figure 6c), with negligible contribution from grain boundary and interfaces. Figure 7f presents corresponding equivalent circuit consists of a single Randles circuit connected in series with *R*_S_″. Notably, the absence of the low‐frequency arc in compliance‐free regime aligns with the disappearance of the nonzero crossing capacitive feature in *I* *–* *V* characteristics at higher *I*_CC_, as shown in Figure S6, Supporting Information, where increasing *I*_CC_ progressively reduces capacitive reactance at a given bias.^[^ ^84^ ^]^ The drop in series resistance by up to two orders of magnitude in the LRS of the compliance‐free regime relative to the HRS (Table S2, Supporting Information) further supports the enhanced conductance arising from intrawire π–π charge transport under higher current conditions. **Table** **1** highlights a summarized depiction of how variations in current modulate the underlying conduction mechanisms, leading to distinct RS regimes. Moreover, EIS analysis reveals that none of the LRS states are purely resistive. This observation contrasts with previous reports on N‐CuMe_2_Pc nanowire‐mesh memristors, where LRS is dominated by Ohmic conduction, attributing the electrochemical metallization.^[^ ^30^ ^]^ Instead, the present study demonstrates a combined resistive and capacitive characteristic, confirming the absence of fully metallic (Ag) or Ohmic filament formation.^[^ ^81^, ^85^ ^]^ This again reinforces the conclusions drawn from the *I* *–* *V* analysis, showing no indication of Ohmic conduction in the LRS under either compliance‐limited or compliance‐free regimes. Nevertheless, direct evidence of Ag^+^‐cation migration through the exterior of the nanowires within the network is beyond the scope of our study.

**Table 1:** Compliance current‐dependent transition from digital to analog switching regimes.

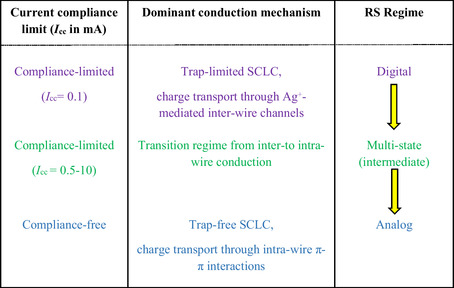		

Moreover, the convergence of complementary techniques, *I* *–* *V*, XPS, and EIS, provides rigorous and robust validation of the proposed conduction mechanisms, establishing a new standard in the structural and functional understanding of nanowire‐network memristive systems.

## 3 Conclusions

This work demonstrates, F_16_CuPc nanowire network is a versatile memristive platform capable of forming‐free multifunctional memory applications. A systematic modulation of the *I*_CC_ enables controlled transitions between distinct RS modes within a single device. At low *I*_CC_, the device exhibits digital switching with an ON/OFF ratio exceeding ≈400. Increasing *I*_CC_ from 0.5–10 mA induces multilevel low‐resistance states, initially reducing the ON/OFF ratio to ≈23, which then restores to digital‐like levels with further current increase. This is followed by a reliable emulation of stable bio‐synaptic behaviors such as potentiation and depression. Mechanistically, this evolution in RS behavior is triggered by a transition from trap‐limited SCLC in the compliance‐limited regime to trap‐free SCLC in the compliance‐free regime. This transition induces a modulation in the dominant conduction pathway, from Ag^+^‐cation‐assisted interwire transport via Ag–F/ Ag–π interactions to intrawire conduction through π–π/ F–π interactions within the nanowires themselves. The results from impedance spectroscopy strongly corroborate the insights obtained from *I* *–* *V* measurements, highlighting a coherent and comprehensive validation of the proposed conduction mechanisms. The novelty of this work is rooted in the controlled growth of nanowire structures through self‐assembled 2D molecular stacking, which plays a pivotal role in enabling the coexistence of multiple RS phenomena, spanning digital, multilevel, and analog RS within a single device architecture. This versatility paves the way for the development of adaptable, energy‐efficient, and cost‐effective organic molecule‐based wearable smart memory technologies, with potential applications in brain‐machine interfaces, robotics, data storage, wearable healthcare devices, and smart textiles.

## 4 Experimental Section

4.1

4.1.1

##### Materials

Copper (II) hexadecafluoro‐phthalocyanine (C_32_CuF_16_N_8_) (80%, average Mw 863.92), isopropanol, and acetone are purchased from Sigma Aldrich and used without further purification. Highly doped p‐type Si wafer (Thickness ≈ 0.5 mm, Resistivity ≈ 0.001–0.05 Ω cm, Crystal Orientation <100>) is purchased from Y Mart, INC. Silver (Ag) conductive paste (99 %) is purchased from RS Pro.

##### Methods

*Substrate Preparation*: Highly doped p‐type Si wafer (*p* ^++^‐Si) substrates are cleaned sequentially in acetone, and isopropanol in an ultrasonic bath for 15 min in each solvent. Next, all substrates are dried with a hot air gun, followed by UV‐ozone treatment for another 15 min before immediate deposition.

*PVT* *‐Synthesis of F*_*16*_ *CuPc Nanowires and Fabrication of Device*: F_16_CuPc powder (7 g) is loaded in a ceramic boat, which is then located at the center of a quartz tube (three‐inch diameter). The quartz tube is then placed in a furnace (Carbolite Gero) as the boat containing precursors in the quartz tube to be centered in the furnace to collect F_16_CuPc nanowires. The quartz tube is thoroughly flushed with Ar gas before the temperature increases to remove ambient gases. The synthesis of F_16_CuPc nanowire is performed by heating up precursor powder at 495 °C above room temperature with a ramping rate of 5 °C min^−1^ at 1.2 Torr pressure and then deposited for 1.5 h under the Ar flow (45 standard cubic centimeters per minute (sccm)) on precleaned *p* ^++^‐Si substrates which are placed at 11 cm far from the precursor position inside the quartz tube. It is noteworthy that the precursor‐to‐substrate temperature gradient of ≈250 °C is maintained during the synthesis. Based on the previous literature, the deposition conditions using the PVT process are optimized to yield an interconnected nanowire network morphology on *p* ^++^‐Si substrate. Next, Ag paste is used to serve as the electrode and to secure copper wires for external electrical connections.

##### Characterization Techniques

*Characterization of* *PVT* *‐Grown Nanowire Network‐based Memristor*: The surface morphology is examined with Carl Zeiss, SUPRA GEMINI55 FESEM system. Cross‐sectional imaging is carried out using a JEOL JSM IT 800 SHL FESEM set up. TEM images are obtained using a JEOL JEM F200 microscope operating at 200 kV. The XPS measurements have been performed with an Axis Supra spectrometer (KRATOS ANALYTICAL‐ make) using a monochromatized X‐ray source (Al, h*ν* = 1486.6 eV). The XRD data are obtained with a Bruker D8 Advance diffractometer.

*I* *–* *V Characterization of the Memristor*: *I–V* characterizations of the devices are performed in the ambient environment using a Keithley 2400 source meter controlled by a KickStart program.

*Impedance Spectroscopy*: Impedance spectra of devices are collected using an impedance module on an electrochemical unit from ZIVE SP1 potentiostat/galvanostat in the ambient frequency range from 10^6^ Hz– 0.1 Hz with a *V*_ac_ amplitude of 10 mV. The data are simulated by Powell's algorithm using a free software “EIS spectrum analyzer.”

## Supporting Information

Supporting Information is available from the Wiley Online Library or from the author.

## Conflict of Interest

The authors declare no conflict of interest.

## Supporting information

Supplementary Material

## Data Availability

The data that support the findings of this study are available from the corresponding author upon reasonable request.
