# Supplementary material for: Versatile Metal Phthalocyanine‐Based Memristive Nanowire Network: Unraveling the Dynamics of Digital to Analog Switching
Source: Small Sci. 2026 Jan 28;6(1):e202500424. doi: 10.1002/smsc.202500424 (PMC12853393; doi:10.1002/smsc.202500424)
Supplement: Supplementary file 1 — Supplementary Material [file SMSC-6-e202500424-s001.pdf]

Supplementary Information

Versatile Metal Phthalocyanine-based Memristive Nanowire Network: Unraveling the Dynamics of Digital to Analog Switching

Sudeshna Maity,[1*] *Aparajita Mandal*,[1] *Prabhanjan Pradhan*,[2,3] *Ankita Ghosh*,[1,4] *Dinesh Topwal*,[4,5] *Biplab K. Patra*,[2,3] *Tapobrata Som*[1,4 *]

[1] SUNAG Laboratory, Institute of Physics, Sachivalaya Marg, Bhubaneswar, 751005, India

[2] Materials Chemistry & Interfacial Engineering Department, CSIR-Institute of Minerals and Materials Technology, Bhubaneswar, 751013, India

[3] Academy of Scientific and Innovative Research (AcSIR), Ghaziabad 201002, India

[4] Homi Bhabha National Institute, Training School Complex, Anushakti Nagar, Mumbai, 400085, India

[5] Condensed Matter and Material Physics Group, Institute of Physics, Sachivalaya Marg, Bhubaneswar, 751005, India

Table S1. Comparative chart of memristive properties in metal phthalocyanines. (NR stands for not reported)

**Device structure**	**Active layer morphology**	**Memristive characteristics**	**SET/RESET (V)**	**On/Off ratio**	**Conduction mechanism behind RS behaviour**	**Ref:**
ITO/ $F_{16}CuPc$ /Al	Thin film	Synaptic behaviour	NR	NR	Proton conduction mechanism	1
ITO/ClCuPc/Al	Thin film	Synaptic behaviour	NR	NR	O^{2-} ions migration mediated mechanism	2
ITO/N-$CuMe_2Pc$/Ag	Nanowire network	RS hysteresis	0.5 V/-1.4 V	10^3	Electrochemical metallization	3
Si/AuNP/CuPc/Au (or Al) And Si/AuNP/CuPc/Hg	NR	WORM And rewritable	~ (±) 1 V And ~ (±) 2 V	10^5-10^6	Trap induced mechanisms	4
ITO/CuPc/Al	NR	Synaptic behaviour	~ (±) 10 V	NR	Trap induced mechanisms	5
ITO/PEDOT:PSS/BP2T/ZnPc/ $F_{16}CuPc$/Au	Thin film	RS hysteresis	(±) 2.5 V	20	Trap induced mechanisms	6
Au/FePc/Au	Thin film	RS hysteresis	< (±) 10 V	Reduced from ~19 to 1.4 after 400 s	Chemisorbed oxygen mediate trapping/de-trapping mechanisms	7
p^{++}-Si/ $F_{16}CuPc$ /Al	Nanowire network	Digital, multilevel, and synaptic behaviour	+ 3 V/ - 1 V	> 400	Migrated Ag^+ cation-mediated conduction, along with intrinsic intra-wire intermolecular trapping/de-trapping mechanisms	This work

Table S2. List of equivalent circuit parameters obtained by fitting impedance spectra. Abbreviation of all the parameters corresponds their respective resistive states. (CC stands for compliance current and DA stands for disappeared.)

**Resistive switching (RS) state**	**Equivalent circuit parameters**									
	**R_s [Ω]**	**R_1 [Ω]**	**R_2 [Ω]**	**R_3 [Ω]**	**Q_1 [nF]**	**α_1**	**Q_2 [nF]**	**α_2**	**Q_3 [nF]**	**α_3**
**Initial RS (IRS)**	17×10^3	0.7×10^6	10^6	0.76×10^6	3.1	0.82	0.19×10^3	0.75	16.9	0.75
**HRS (at CC-limited V_{RESET})**	11×10^3	0.6×10^6	10^6	0.84×10^6	2.74	0.84	0.18×10^3	0.74	13.7	0.76
**HRS (at CC-free V_{RESET})**	1.5×10^3	0.5×10^6	10^6	0.86×10^6	1.9	0.89	0.17×10^3	0.77	12.2	0.78
**LRS (at CC-limited V_{SET})**	6.6×10^3	0.77×10^6	0.1×10^6	DA	1.2	0.84	2.25×10^3	0.73	DA	DA
**LRS (at CC-free V_{SET})**	80.57	16×10^3	DA	DA	1.6	0.81	DA	DA	DA	DA

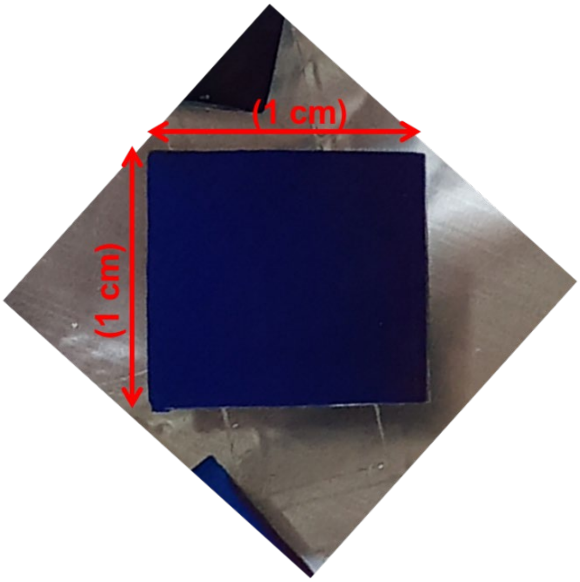

Figure S1. Image of *in*-situ physical vapour transport-grown $F_{16}CuPc$ sample.

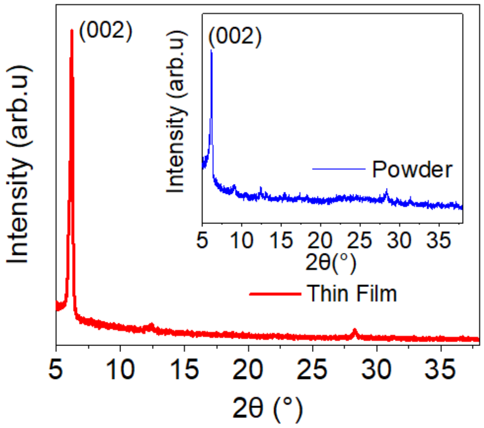

Figure S2. XRD pattern PVT grown $F_{16}CuPc$ thin film on p^{++}-Si substrate and on as received precursor powder (Inset).

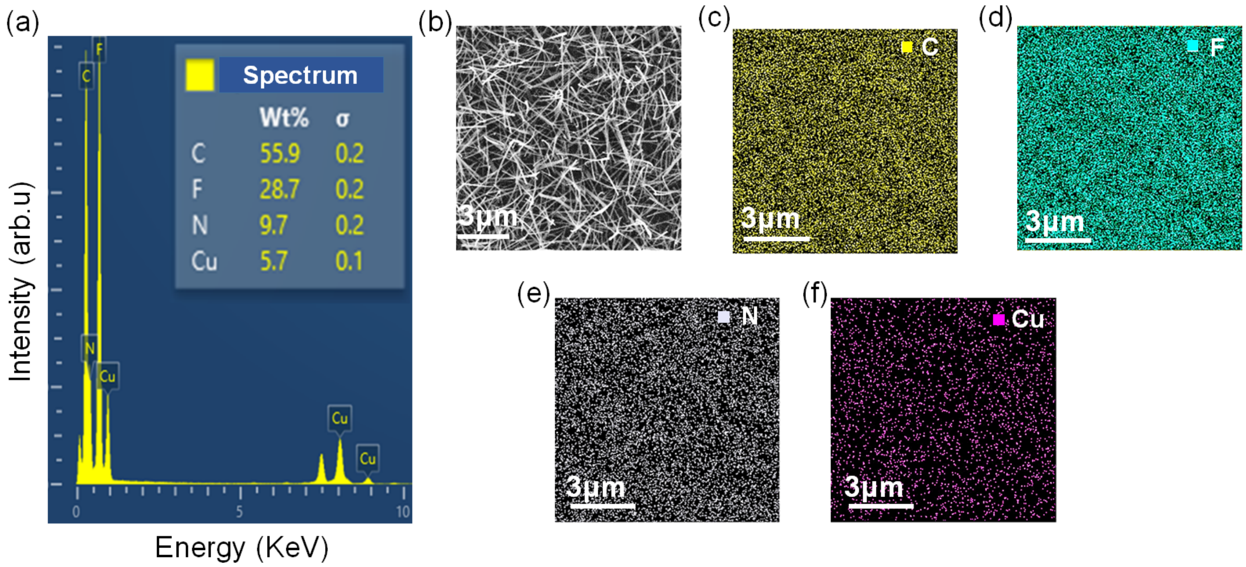

Figure S3. (a) EDX spectrum of $F_{16}CuPc$ along with the atomic weight percentages. (b-f) EDX mapping of $F_{16}CuPc$ performed in TEM.

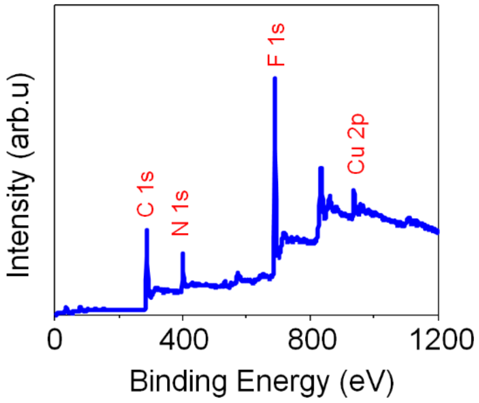

Figure S4. XPS survey spectra of as-grown $F_{16}CuPc$ film in the ambient atmosphere with the indication of significant peaks.

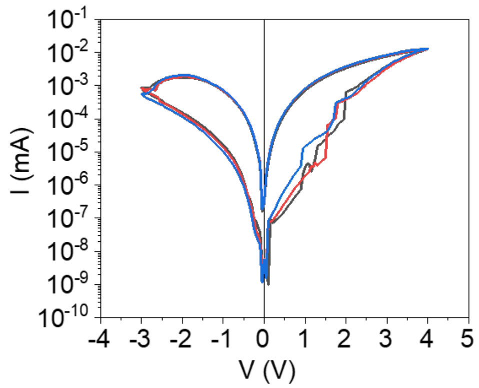

Figure S5. Semi-log *I-V* characteristics in the compliance-free regime obtained by increasing I_{CC} beyond 10 mA.

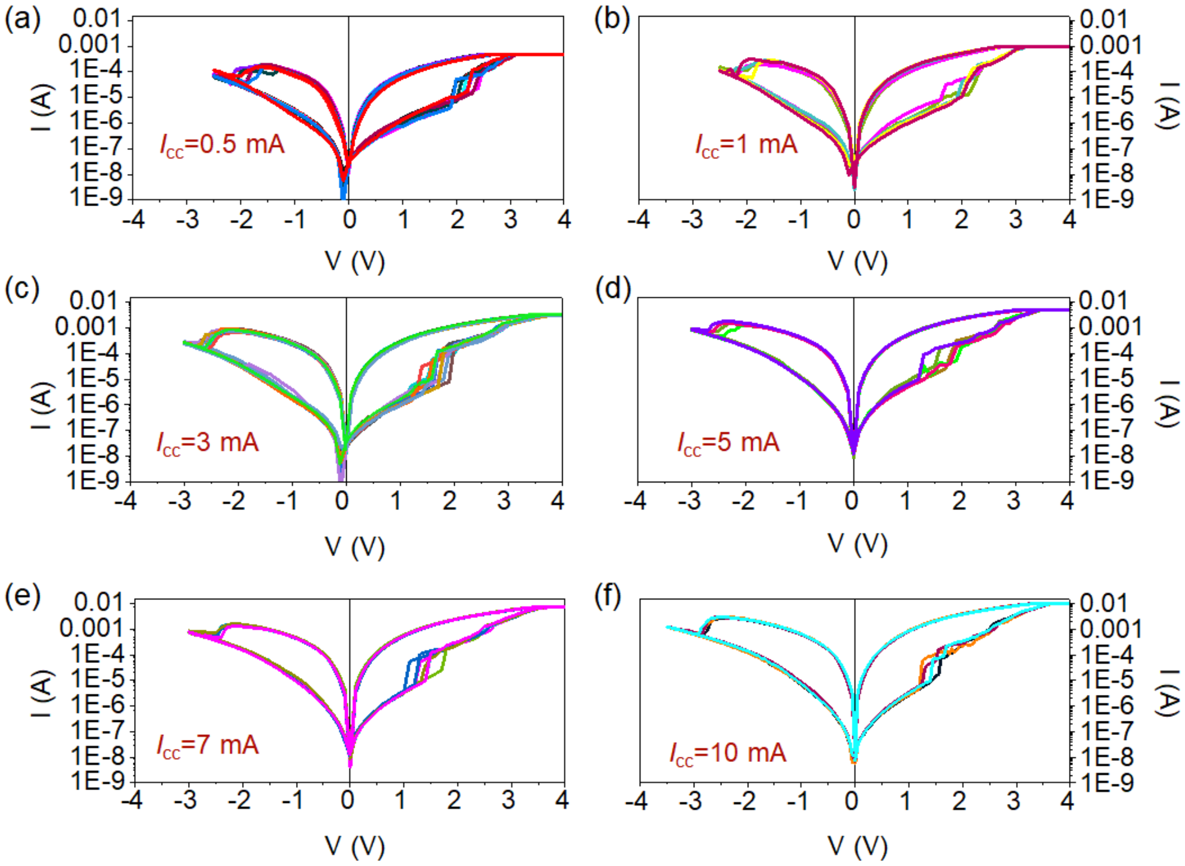

Figure S6. (a)-(f) Individual semi-log *I-V* plots for the clearer view of the disappearance of non-zero crossing pure capacitive feature at $I_{CC} \geq 5$ mA.

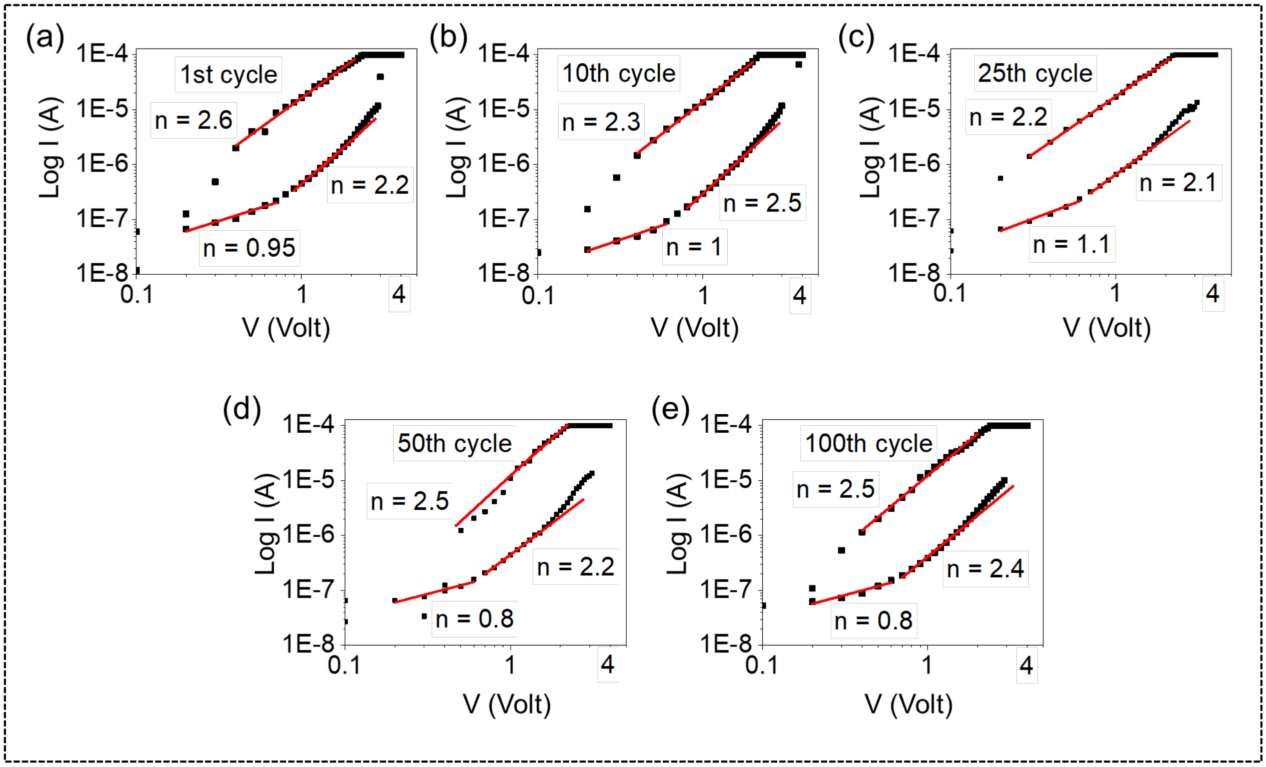

Figure S7. (a)-(d) **Cycle-to-cycle** variation of the extracted slopes for compliance-limited regime. For clarity, five representative *I–V* cycles with the corresponding slope (n) values are shown from a total set of 100 cycles. The red solid lines represent corresponding linear fitting of the curves.

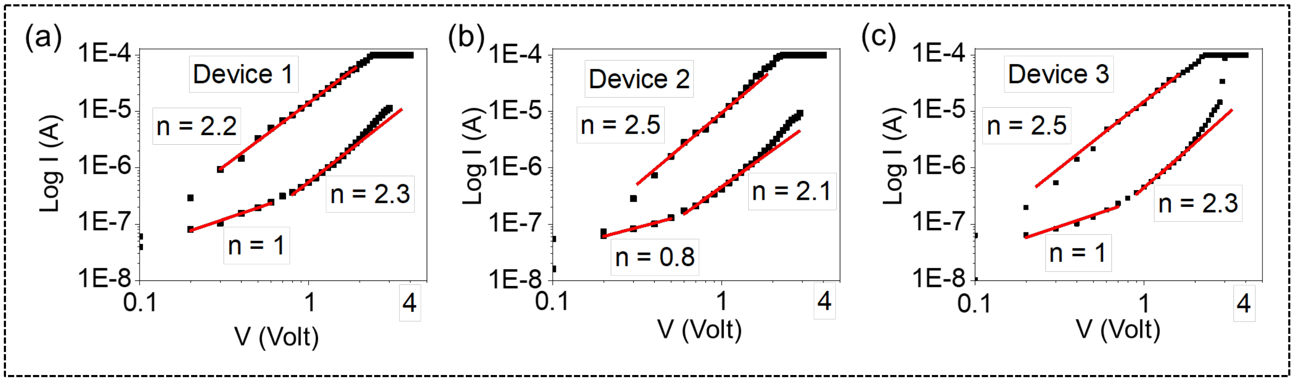

Figure S8. (a)-(c) **Device-to-device** variation of the extracted slopes for compliance-limited regime. For clarity, *I-V* cycles from three representative devices with the same configuration (as discussed in the manuscript) are presented with the corresponding slope (n) values. The red solid lines represent corresponding linear fitting of the curves.

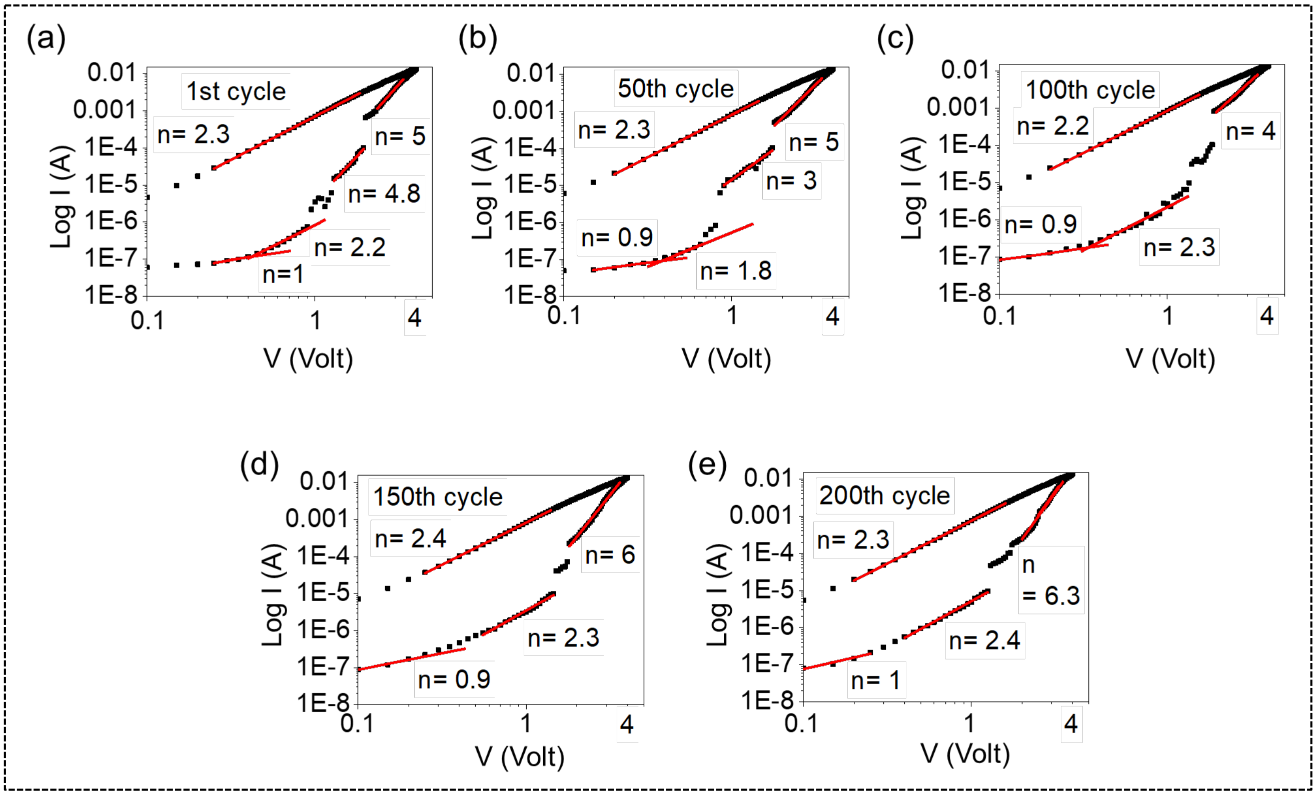

Figure S9. (a)-(e) **Cycle-to-cycle** variation of the extracted slopes for compliance-free regime. For clarity, five representative *I–V* cycles with the corresponding slope (n) values are shown from a total set of 200 cycles. The red solid lines represent corresponding linear fitting of the curves.

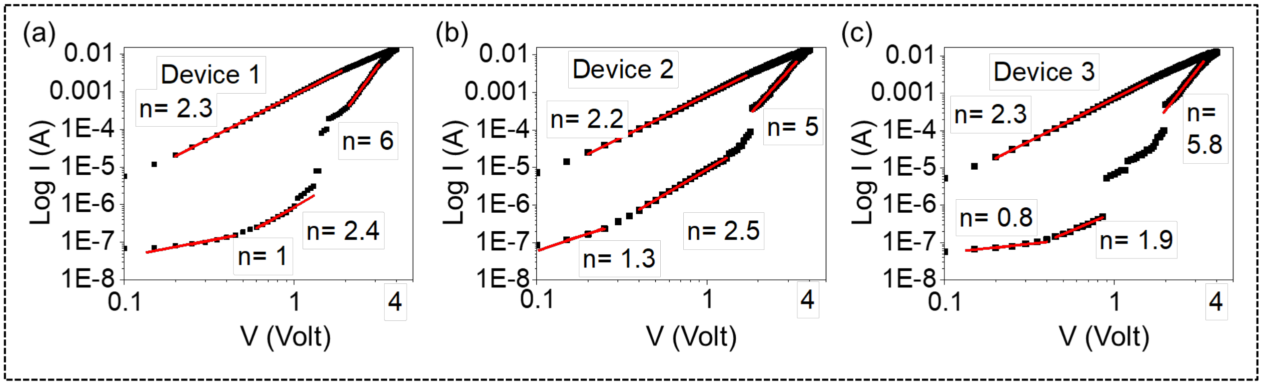

Figure S10. Device-to-device variation of the extracted slopes for compliance-free regime. For clarity, *I-V* cycles from three representative devices with the same configuration (as discussed in the manuscript) are presented with the corresponding slope (n) values. The red solid lines represent corresponding linear fitting of the curves.

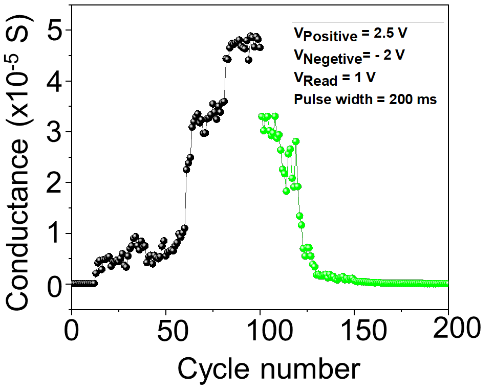

Figure S11. Potentiation/depression behaviour in low I_{CC} regime.

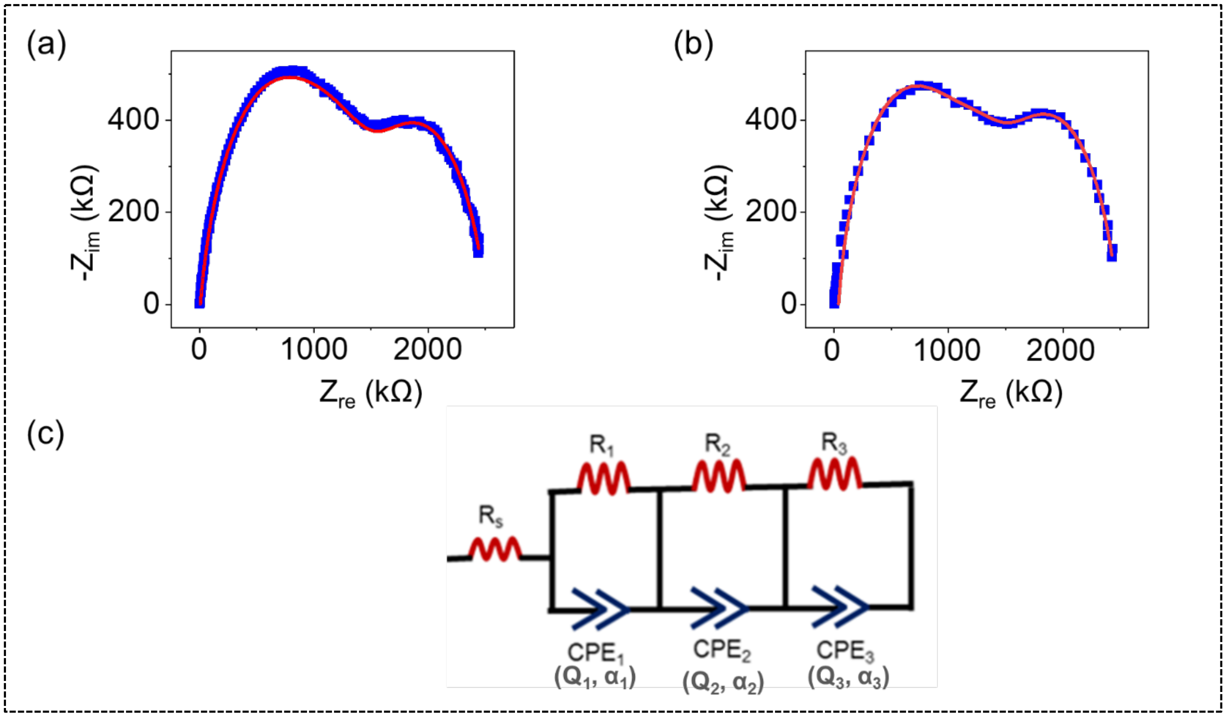

Figure S12. (a–b) Nyquist plots at **HRS** for two different cycles (1st and 5th) on a **test device** using the same configuration as discussed in the manuscript. The solid lines represent the fitted curves obtained using Powell's algorithm. (c) Equivalent circuit used for the fitting the curves.

Table S3: Equivalent circuit fitting parameters at **HRS state** for different cycle on the **test device**.

RS state	Equivalent circuit parameters									
	R_s [Ω]	R_1 [Ω]	R_2 [Ω]	R_3 [Ω]	Q_1 [nF]	α_1	Q_2 [nF]	α_2	Q_3 [nF]	α_3
**HRS (Initial cycle)**	6×10^3	0.7×10^6	10^6	0.83×10^6	3.2	0.8	0.19×10^3	0.75	21.2	0.75
**HRS (Last cycle)**	21×10^3	10^6	10^6	0.45×10^6	2.6	0.83	0.17×10^3	0.77	29.5	0.85

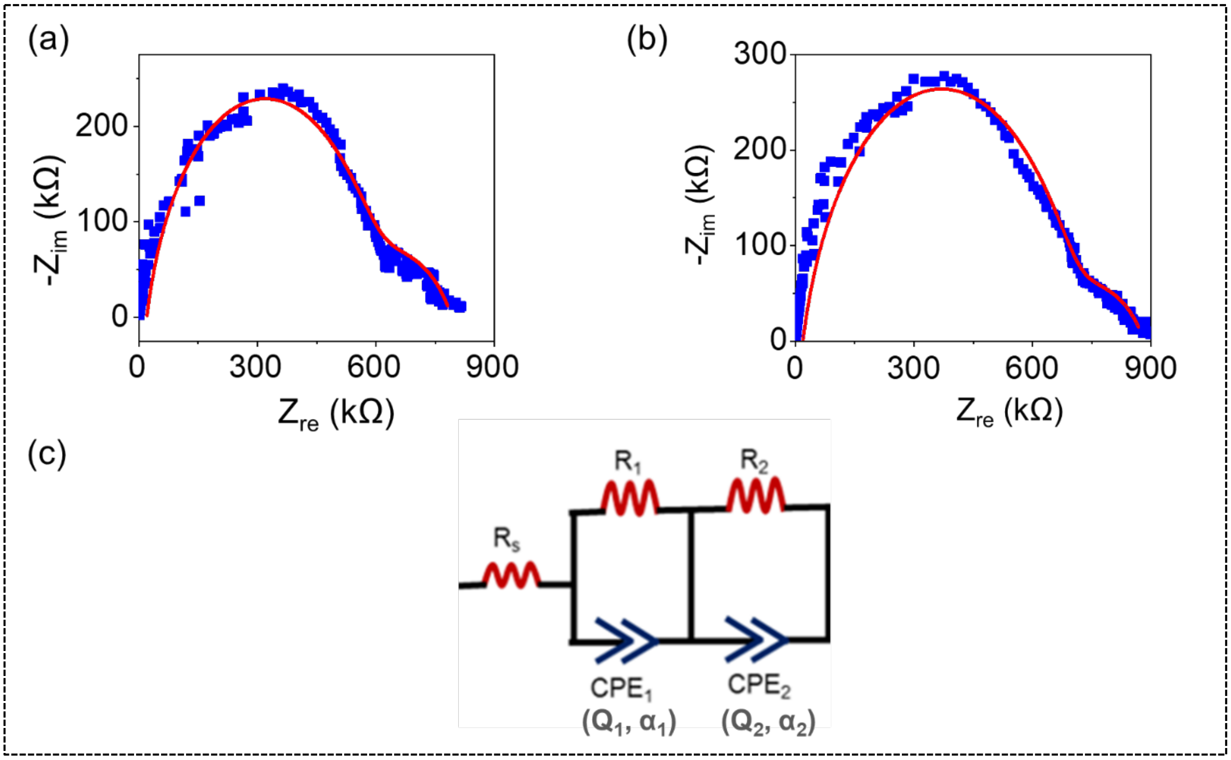

Figure S13. (a)-(b) Nyquist plots at LRS in compliance-limited regime for two different cycles (1st and 5th) on a **test device** using the same configuration as discussed in the manuscript. The fitted curves (solid lines) are achieved using Powell's algorithm. (c) Equivalent circuit used for the fitting the curves.

Table S4: Equivalent circuit fitting parameters at **compliance current-limited LRS state** for different cycle on the **test device**.

RS state (Compliance-limited)	Equivalent circuit parameters						
	R_s [Ω]	R_1 [Ω]	R_2 [Ω]	Q_1 [nF]	α_1	Q_2 [nF]	α_2
**LRS (1st cycle)**	11×10^3	0.6×10^6	0.2×10^6	1.2	0.84	0.4×10^3	0.75
**LRS (5th cycle)**	15×10^3	0.7×10^6	0.2×10^6	1.4	0.83	0.8×10^3	0.78

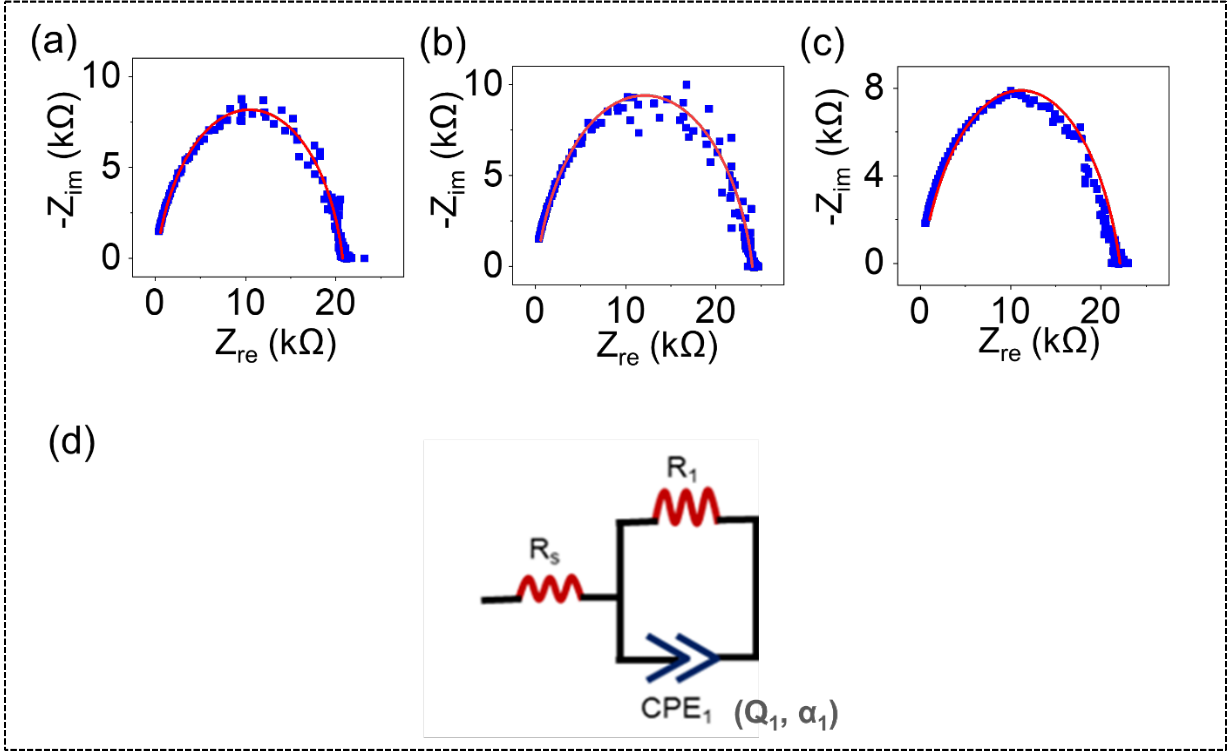

Figure S14. (a)-(c) Nyquist plots at LRS in compliance-free regime for three different cycles (1st, 5th and 10th) on a **test device** using the same configuration as discussed in the manuscript. The fitted curves (solid lines) are achieved using Powell's algorithm. (d) Equivalent circuit used for the fitting the curves.

Table S5: Equivalent circuit fitting parameters at **compliance current-free LRS state** for different cycle on **test device**.

RS state (Compliance-free)	Equivalent circuit parameters			
	R_s [Ω]	R_1 [Ω]	Q_1 [nF]	α_1
LRS (1st cycle)	1.7×10^2	20×10^3	1.02	0.85
LRS (5th cycle)	1.7×10^2	23×10^3	1.1	0.85
LRS (10th cycle)	1.8×10^2	21×10^3	1.7	0.8

Reference:

1. J. Li, Y. Qian, W. Li, Y. H. Lin, H. Qian, T. Zhang, K. Sun, J. Wang, J. Zhou, Y. Chen, J. Zhu, G. Zhang, M. Yi, W. Huang, *Adv. Electron. Mater.* **2022**, *8*, 1.
2. J. Zhou, W. Li, Y. Chen, Y. H. Lin, M. Yi, J. Li, Y. Qian, Y. Guo, K. Cao, L. Xie, H. Ling, Z. Ren, J. Xu, J. Zhu, S. Yan, W. Huang, *Adv. Mater*. **2021**, *33*, 1.
3. Z. Lv, Q. Hu, Z. X. Xu, J. Wang, Z. Chen, Y. Wang, M. Chen, K. Zhou, Y. Zhou, S. T. Han, *Adv. Electron. Mater*. **2019**, *5*, 1.
4. N. Padma, C. A. Betty, S. Samanta, A. Nigam, *J. Phys. Chem. C* **2017**, *121*, 5768.
5. L. Wang, Z. Wang, J. Lin, J. Yang, L. Xie, M. Yi, W. Li, H. Ling, C. Ou, W. Huang, *Sci. Rep.* **2016**, *6*, 1
6. Q. Meng, X. He, Q. Mao, Y. Weng, J. Yang, D. Yan, H. Zhao, *Appl. Phys. Lett*. **2015**, *106*, 173302.
7. S. Samanta, A. Singh, A. K. Debnath, D. K. Aswal, S. K. Gupta, J. V. Yakhmi, S. Singh, S. Basu, S. K. Deshpande, *J. Appl. Phys.* **2008**, *104*, 073717.
